# Engineered Substrate‐Free Small Molecules for Enhanced Raman Scattering and Photothermal Conversion Efficiency

**DOI:** 10.1002/advs.202505467

**Published:** 2025-09-05

**Authors:** Sheng Yu, Shuai Gao, Yongming Zhang, Sihang Zhang, Jingwen Sun, Wenxian Zhang, Tingting Li, Kai Cui, Zeyu Xiao, Wei Lu

**Affiliations:** ^1^ School of Pharmacy, Minhang Hospitial, Key Laboratory of Smart Drug Delivery, Ministry of Education, State Key Laboratory of Molecular Engineering of Polymers Fudan University 826 Zhangheng Road Shanghai 201203 China; ^2^ Department of Pharmacology and Chemical Biology Institute of Molecular Medicine Shanghai Jiao Tong University School of Medicine 280 South Chongqing Road Shanghai 200025 China; ^3^ Hangzhou Institute of Medicine (HIM) Chinese Academy of Sciences 150 East Street Hangzhou Zhejiang 310022 China; ^4^ Quzhou Fudan Institute 108 Minjiang Avenue Quzhou Zhejiang 324002 China

**Keywords:** in vivo Raman imaging, molecular engineering, photothermal therapy, Raman scattering, theranostics

## Abstract

Raman spectroscopy with surface‐enhanced Raman scattering (SERS) through metal substrates is a highly precise bioimaging technique. Alternatively, recently discovered small molecules to enhance the Raman signal intensities through their self‐stacking, termed stacking‐induced intermolecular charge transfer‐enhanced Raman scattering (SICTERS), offer ultrasensitive in vivo Raman imaging free of substrates. Molecular engineering to increase the SICTERS intensity and to tune photothermal conversion efficiency of these molecules is critical for furthering their biomedical application but not yet feasible. Here, by increasing the length of side chain and introducing the benzene ring to bis‐thienyl‐substituted benzobisthiadiazole, this study demonstrates an optimized molecule BBTPPRO that possesses both high SICTERS intensity and photothermal conversion efficiency (31.19%). The prepared BBTPPRO nanoparticles achieve intraoperative Raman image‐guided photothermal therapy (PTT) of orthotopic mouse colon tumor. Overall, this report presents a molecular strategy combing the principle of SICTERS with the Jablonski diagram to design substrate‐free Raman small molecules toward SICTERS‐mediated photo‐theranostic agents.

## Graphical abstract

A molecular engineering strategy is developed to increase the stacking‐induced intermolecular charge transfer‐enhanced Raman scattering (SICTERS) intensity and photothermal conversion efficiency of electron donor‐acceptor‐donor (D–A–D) molecules. This approach enables to design SICTERS‐mediated photo‐theranostic agents for substrate‐free Raman image‐guided cancer photothermal therapy.

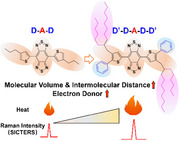

## 1 Introduction

Raman spectroscopy represents an ultrasensitive imaging modality for chemical analysis, environmental sciences, and biomedical application.^[^ ^1^, ^2^, ^3^, ^4^ ^]^ But spontaneous Raman signal is very weak for detection.^[^ ^5^, ^6^, ^7^ ^]^ The development of techniques and strategies such as surface‐enhanced Raman scattering (SERS), resonance Raman scattering (RRS), coherent anti‐Stokes Raman scattering (CARS), and stimulated Raman scattering (SRS) enables the amplification of Raman probe signals.^[^ ^8^ ^]^ SERS that can reach the enhancement factor as high as ≈10^10^ affords high specificity and sensitivity for detections in vivo.^[^ ^9^, ^10^, ^11^, ^12^ ^]^ However, SERS relies on metal substrates that bring the issues of poor biocompatibility and the biosafety concerns for biomedical applications.^[^ ^13^, ^14^ ^]^ In RRS, the energy of the excitation is adjusted to overlap with an electronic transition of the molecule of interest, which results in significant amplification of scattered light. The phototoxicity caused by shorter excitation wavelengths limits the application of RRS.^[^ ^15^ ^]^ Additionally, the enhancement factor of RRS is not large enough to generate a detectable signal for in vivo imaging.^[^ ^16^ ^]^ SRS or CARS is a nonlinear optics‐based Raman process that relies on two synchronized laser pulses (pump light and Stokes light) to gain signal amplification through optical coherence and vibrational resonance.^[^ ^17^, ^18^ ^]^ Due to a nonresonant background, CARS spectrum is different from its corresponding spontaneous Raman spectrum, which causes difficulties in image interpretation, and limits the detection sensitivity.^[^ ^18^ ^]^ SRS doesn't have a nonresonant background and could realize in vivo Raman imaging using highly active Raman tags such as alkynyl, cyano, and C─D bonds.^[^ ^19^, ^20^, ^21^ ^]^ The high cost of instruments, complicated operation, and limited spectral information hinder in vivo application of SRS technology.

Alternatively, we have recently reported a class of small‐molecule Raman reporters with planar bis‐thienyl‐substituted benzobisthiadiazole structure.^[^ ^22^ ^]^ The molecules through conjugated electron donor–acceptor–donor (D–A–D) system increase the electron delocalization and lower the energy gap, thus facilitating the intramolecular charge‐transfer excitations. Moreover, the molecules can pile up to allow the intermolecular charge‐transfer from one molecule to the neighboring molecule in both in‐plane and out‐of‐plane directions, leading to 3D charge‐transfer. As a result, the stacked molecules greatly improve the Raman scattering cross‐section through a mechanism named stacking‐induced charge transfer‐enhanced Raman scattering (SICTERS). Compared with SERS, the SICTERS nanoprobes enhance the Raman scattering without the need for those inorganic substrates, which can avoid the associated biocompatibility concerns and show great potential for clinical translation. Despite the successful in vivo Raman imaging, the underlying relationship between the molecular structure and the SICTERS intensity remains elusive.

Traditional imaging modalities such as magnetic resonance imaging (MRI) and computed tomography (CT) provide whole‐body imaging, facilitating preoperative tumor diagnosis. However, due to the ionizing radiation hazards, CT cannot be used for intraoperative imaging.^[^ ^23^ ^]^ Intraoperative MRI is high‐cost and complexity of operation, only available in large medical centers.^[^ ^24^ ^]^ By contrast, optical imaging techniques such as fluorescence imaging and Raman imaging enable real‐time image acquisition with high spatial resolution, providing a super‐solution intraoperative images to guide surgical resection or phototherapy.^[^ ^25^, ^26^ ^]^ In recent years, theranostic nanoparticles for image‐guided tumor photothermal therapy (PTT) have attracted increasing attention. Inorganic photothermal agents such as gold‐based nanomaterials and carbon‐based nanomaterials have an excellent light‐to‐heat conversion capability, but these inorganic PTT agents have been limited in their clinical application due to their slow or non‐biodegradability and potential long‐term toxicity. Alternatively, small organic agents have better biocompatibility and can be designed with multiple functions, including imaging and PTT capabilities^[^ ^27^, ^28^ ^]^ Precise PTT under the guidance of fluorescence imaging can realize effective treatment of tumor by using small organic agent such as BPBBT.^[^ ^29^ ^]^ Therefore, taking strategy to modulate the photothermal conversion efficiency of these SICTERS molecules is also critical for the development of SICTERS‐based theranostics.

According to the Jablonski diagram, the fluorescence and photothermal conversion effect of a D–A‐structured molecule can be tuned by balancing the competition between radiative and non‐radiative transition pathways.^[^ ^30^, ^31^ ^]^ Reduction of the highest occupied molecular orbital – lowest unoccupied molecular orbital (HOMO–LUMO) energy gap through D–A engineering red‐shifts the absorption bands to the near‐infrared (NIR) region, and favors light penetration into deeper tissue and stronger light‐to‐heat process.^[^ ^32^, ^33^, ^34^, ^35^ ^]^ Besides, molecular modulation with long alkyl chains that provides space for free intramolecular motions has shown an effective approach to boost heat generation.^[^ ^36^, ^37^ ^]^

**Scheme 1:**
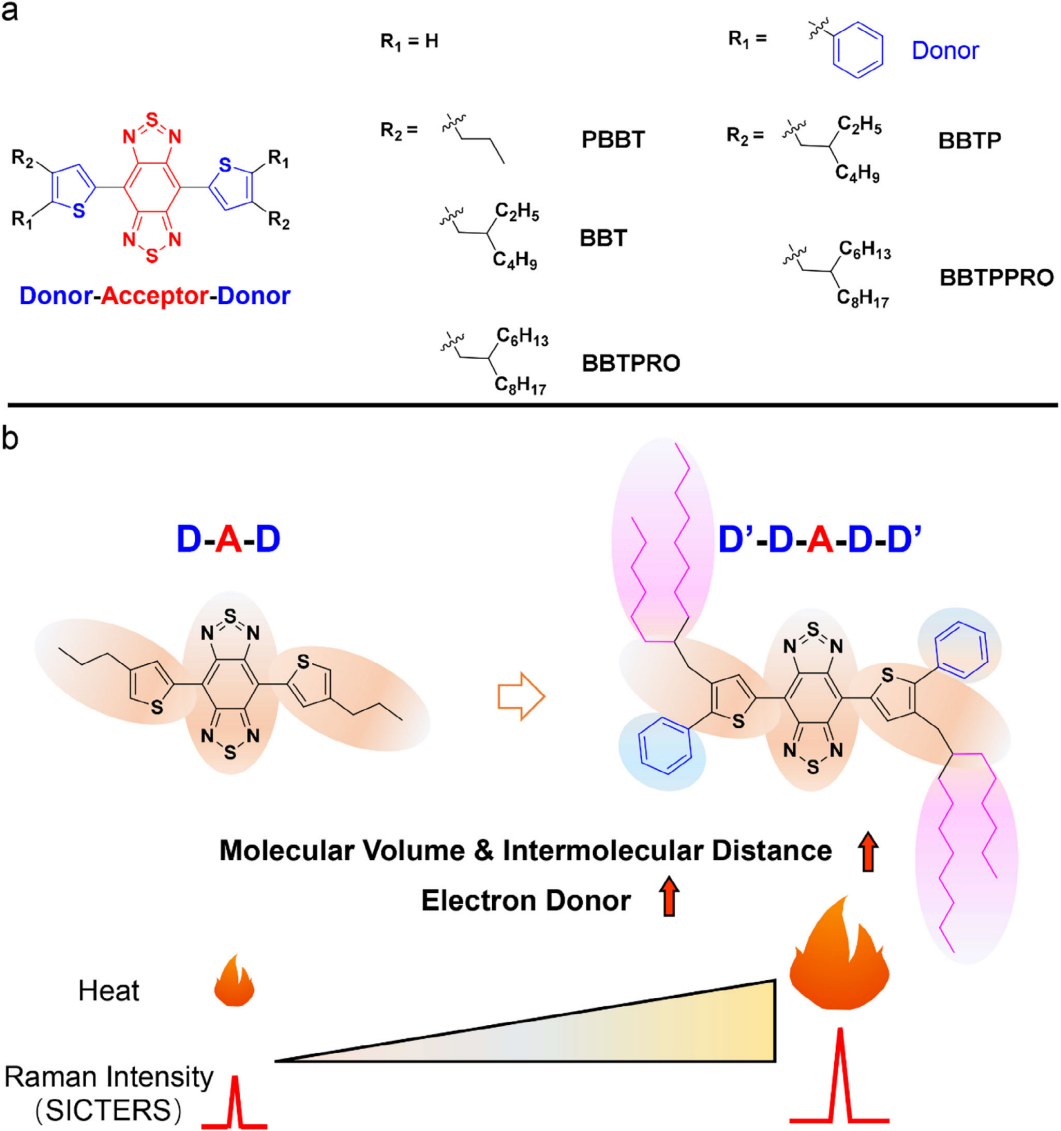
Molecular design of substrate‐free small molecules with enhanced SICTERS and photothermal conversion efficiency. a) Chemical structures of design molecules. b) Scheme of the molecular structures and optical physical properties.

Here, we hypothesize that the increase in molecular volume and electron donor of a substrate‐free planar D–A–D molecule improves both its SICTERS intensity and photothermal conversion efficiency. As a proof‐of‐concept, alkyl side chains with different lengths and/or phenyl groups are introduced to the lead compound 4,7‐di(thiophen‐2‐yl) benzobisthiadiazole (**Scheme** **1**a). With the increase in the length of side chains and the addition of phenyl groups, the signal intensity of Raman characteristic peaks increases owing to the enhancement of polarizability through enlarging molecular volume and lowering the HOMO–LUMO energy gap. Meanwhile, the photothermal conversion efficiency is elevated, ascribing to the extension of intermolecular distance and the red‐shifted absorption bands (Scheme 1b). Among these compounds, BBTPPRO owns the best Raman signal intensity at 894 cm^−1^ and the highest photothermal conversion efficiency (31.19%) by introducing 2‐hexyldecyl chains and phenyl group to thiophene group. In the orthotopic CT26‐Luc colon tumor mouse model, the intraoperative Raman image‐guided PTT is achieved to completely remove the tumor using the BBTPPRO nanoparticles (NPs) as the SICTERS‐mediated photo‐theranostic agents.

## 2 Results

### 2.1 Molecular Design, Synthesis, and Density Functional Theory Calculation

**Figure 1:**
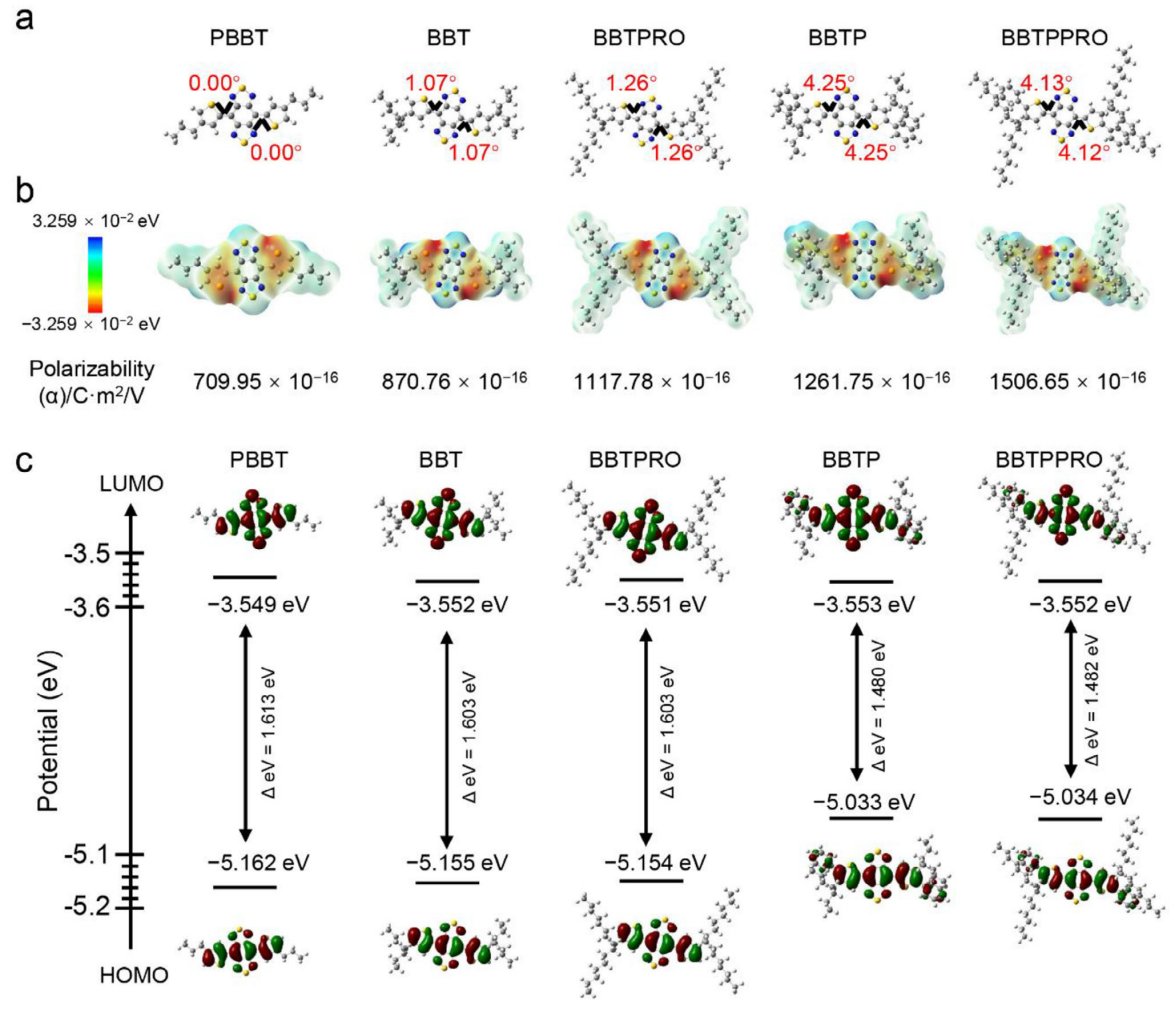
DFT calculation of substrate‐free Raman molecules. a) The optimized ground (S_0_) of molecules with dihedral angles. b) ESP maps of molecules. c) HOMO–LUMO distributions of molecules. The dihedral angles, ESP maps, and HOMO and LUMO with the energy gaps (Δ eV) of the optimized structures at S_0_ were calculated by DFT (Gaussian 09/B3LYP/6‐31G (d)).

A series of substrate‐free organic Raman molecules by introducing Alkyl chain and phenyl group were designed and synthesized based on the plane‐structured molecule of 4,7‐di(thiophen‐2‐yl) benzobisthiadiazole (Scheme 1a). The molecule grafted with a branched long 2‐hexyldecyl group is denoted as BBTPRO. The one with 2‐ethylhexyl is named as BBT, while the one with the linear 1‐propyl unit is named as PBBT. BBT modified with phenyl groups on both sides is denoted as BBTP, while BBTPRO with phenyl groups is denoted as BBTPPRO. The synthesis route is shown in Figure S1 (Supporting Information). The molecular structures of the associated intermediates were characterized and confirmed by ^1^H and ^13^C nuclear magnetic resonance spectra (NMR) as well as time‐of‐flight mass spectra (Figures S2–S8, Supporting Information). The density functional theory (DFT) calculation demonstrated that all the designed molecules were plane‐structured with the dihedral angles between the benzobisthiadiazole core and the thiophene blocks less than 5° (**Figure** **1**a). The grazing‐incidence wide‐angle X‐ray scattering (GIWAXS) spectra showed strong *π‐π* stacking of these Raman molecules in solid state (Figure S9, Supporting Information). The planar conformation resulted in the fluorescence quenching in the aggregate forms of these molecules in the mixture of H_2_O/tetrahydrofuran (THF) when the water content was 60% or more (Figure S10, Supporting Information). The reduced fluorescence may favor non‐radiative decay, i.e., photothermal conversion, and improve the Raman signal‐to‐noise ratio.^[^ ^30^, ^38^ ^]^ The electrostatic potential (ESP) map showed that PBBT, BBT, and BBTPRO were D−A−D‐typed molecules (Figure 1b). Following addition of moderate electron‐donating phenyl groups, BBTP and BBTPPRO became the D′−D−A−D−D′ types, with increased energy level of HOMO and reduced the energy gap between HOMO and LUMO (Figure 1b,c). As a result, BBTP and BBTPPRO in THF displayed a red shift of absorption bands than the other three molecules (Figure S11a, Supporting Information). Comparatively, in the mixture of H_2_O/THF (95:5, v/v), the absorption peaks of all five molecules in aggregated states showed broader absorption bands with longer red‐shift due to exciton coupling‐induced intramolecular charge transfer (Figure S11b, Supporting Information).^[^ ^39^ ^]^

### 2.2 Raman Signal Intensities of Substrate‐Free Raman Molecules

**Figure 2:**
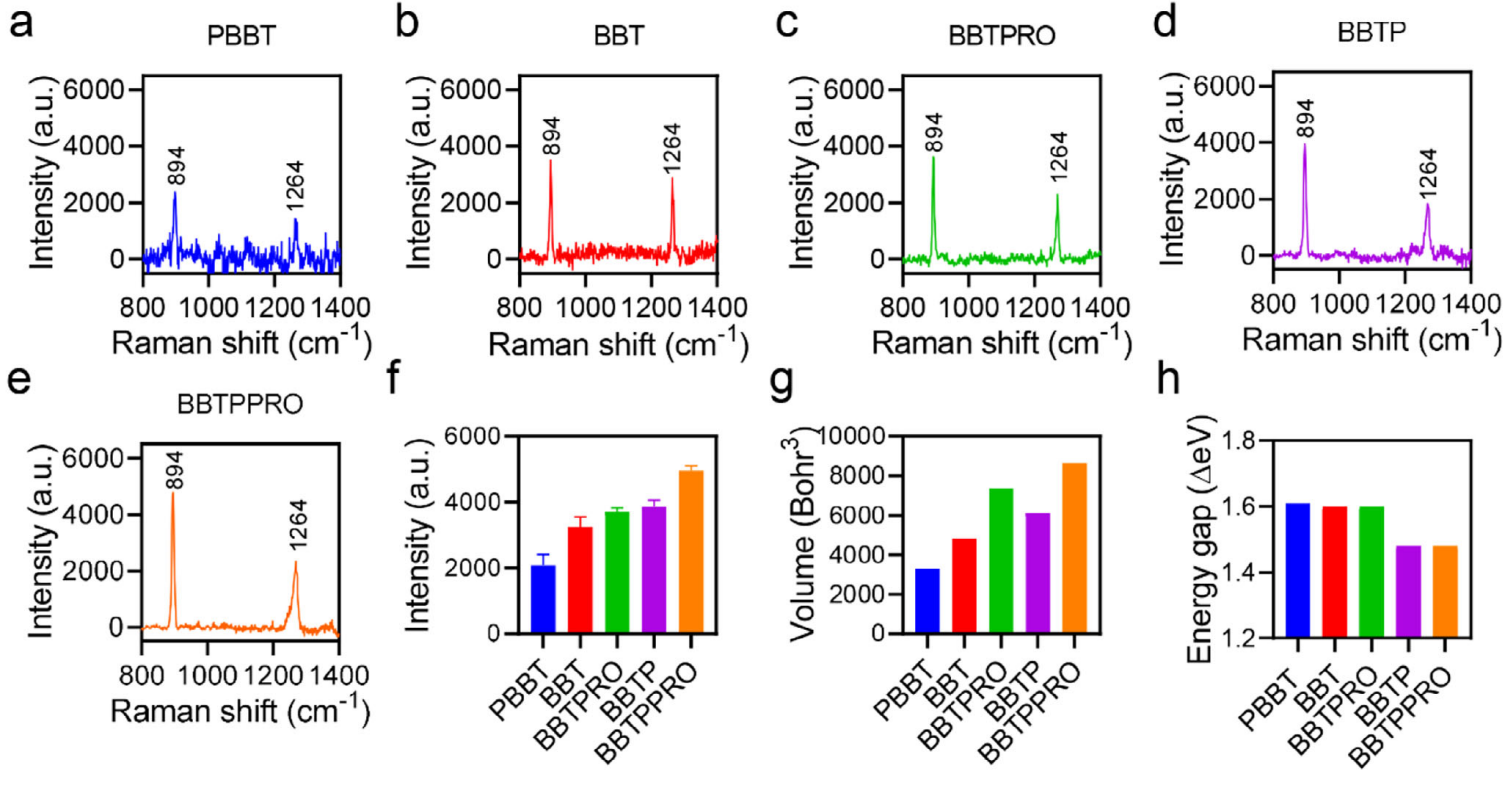
Raman characterization of substrate‐free Raman molecules. a–f) Raman spectra (a–e) and intensity at 894 cm^−1^ (f) of different molecules (10 µm) measured in the mixture of H_2_O/THF (95:5, v/v) with 785‐nm excitation, 5X objective, 1 s of acquisition time, and five times accumulation. g) Van der Waals volume of molecules calculated with Multiwfn software. h) The energy gap (Δ eV) between HOMO and LUMO at S_0_ calculated by DFT (Gaussian 09/B3LYP/6‐31G (d)).

All the Raman molecules in the mixture of H_2_O/THF (95%:5%) had characteristic peaks at 894 and 1264 cm^−1^ (**Figure** **2**a–e). With the increase of length of alkyl chains and the introduction of phenyl groups, the Raman intensity at 894 cm^−1^ was enhanced (Figure 2f). And the increase in the signal‐to‐noise ratio (S/N) at 894 cm^−1^ showed a similar trend to that of the Raman intensity at 894 cm^−1^ of the SICTERS molecules (Figure S12, Supporting Information). BBTPPRO with the longest alkyl side chains and the incorporation of phenyl groups possessed the highest Raman intensity and the highest S/N at 894 cm^−1^ among the five molecules.

Besides, the lipophilic BBTPPRO was dissolved in pure THF to prepare the standalone BBTPPRO molecule. However, the Raman signal of BBTPPRO in pure THF was not detectable due to its substantial fluorescent interference in its molecular form in THF (Figure S13a, Supporting Information). As shown in Figure S13b (Supporting Information), the fluorescence intensity of BBTPPRO was significantly enhanced when the THF fraction in the THF:water mixture was increased to 60% or higher (i.e., water fraction decreased to 40% or lower). This was in line with the disappearance of Raman signal peaks at 894 cm^−1^ of BBTPPRO in the water and THF mixture with the water fraction of 40% or lower (Figure S14a, Supporting Information, orange and purple curves). The characteristic Raman peaks correlated with the degree of aggregation states. Higher degree of aggregation resulted in better signal‐to‐noise ratios of Raman spectral peaks (Figure S14b–d, Supporting Information). In short, the intense Raman peak in Figure 2 occurred only in the aggregated state of BBTPPRO. The Raman signal of BBTPPRO in true solution, such as THF, was not detectable.

The increase in the peak intensity at 894 cm^−1^ was not consistent with that of 1264 cm^−1^, suggesting that alkyl side chains and phenyl groups didn't exert similar effects on the Raman intensity of both peaks (Figure S15, Supporting Information). The results of DFT calculation revealed that the peaks at 894 cm^−1^ of these molecules were mainly assigned to the ring stretchings and bendings of the benzobisthiadiazole core (Movies S1–S5, Supporting Information). The results of vibrational energy distribution analysis (VEDA) further confirmed that this peak was assigned to the stretching of chemical bond S─N and bending of N─C─C of the core (Figures S16–S20, Supporting Information).^[^ ^40^, ^41^ ^]^ With the introduction of benzene rings and the extension of chain length, the enhancement of polarizability can lead to a noticeable increasing trend in the intensity of the peak at 894 cm^−1^. However, the peaks of PBBT, BBT, and BBTP at 1264 cm^−1^ were mostly assigned to different stretching and bending of chemical bonds and in thienyl or other substituted groups (Figures S16,S17, and S19, Supporting Information). The variation of the intensity at 1264 cm^−1^ indicated that the vibration of these assigned chemical bonds may be affected by other factors in addition to polarizability. Compared with the peak at 1264 cm^−1^, the one at 894 cm^−1^ with stronger Raman intensity was selected for further investigation.

Based on electromagnetic theory, the intensity of Raman scattering is proportional to the square of the electric dipole moment (ρ), where ρ is expressed as the product of the molecular polarizability (α) and electric field intensity (E), ρ = αE.^[^ ^42^, ^43^ ^]^ The polarizability is positively correlated with the molecular volume.^[^ ^44^, ^45^ ^]^ Therefore, the longer alkyl chain introduced led to the larger van der Waals volume of the molecule, resulting in higher polarizability.^[^ ^46^, ^47^, ^48^ ^]^ Accordingly, since the polarizability of BBTPRO was greater than that of BBT or PBBT (Figures 1b and 2g), the Raman intensity of BBTPRO was higher than that of BBT or PBBT (Figure 2f). In addition, based on the sum‐over‐state (SOS) formula, polarizability is negatively correlated with the excitation energy, which is related to the energy gap between HOMO and LUMO.^[^ ^49^ ^]^ The introduction of phenyl groups, serving as an electron donor, to the D‐A‐D core formed a stronger D′−D−A−D−D′ system. The results of ESP maps showed substantially increased intramolecular charge‐transfer characteristic and polarizability of BBTP or BBTPPRO compared with BBT or BBTPRO (Figure 1b). The HOMO and LUMO of the two molecules BBTP and BBTPPRO compared with the other three molecules without phenyl groups also demonstrated that the conjugated D′−D−A−D−D′ system increased the extent to electron delocalization and lowered the energy gap, facilitating charge‐transfer excitations (Figures 1c and 2h). In conclusion, besides the molecular volume, the introduction of an electron‐donor benzene ring to form a D′−D−A−D−D′ structure increased electron delocalization and narrowed the energy gap, further enhanced polarizability.

### 2.3 Photothermal Properties of Substrate‐Free Raman Molecules

**Figure 3:**
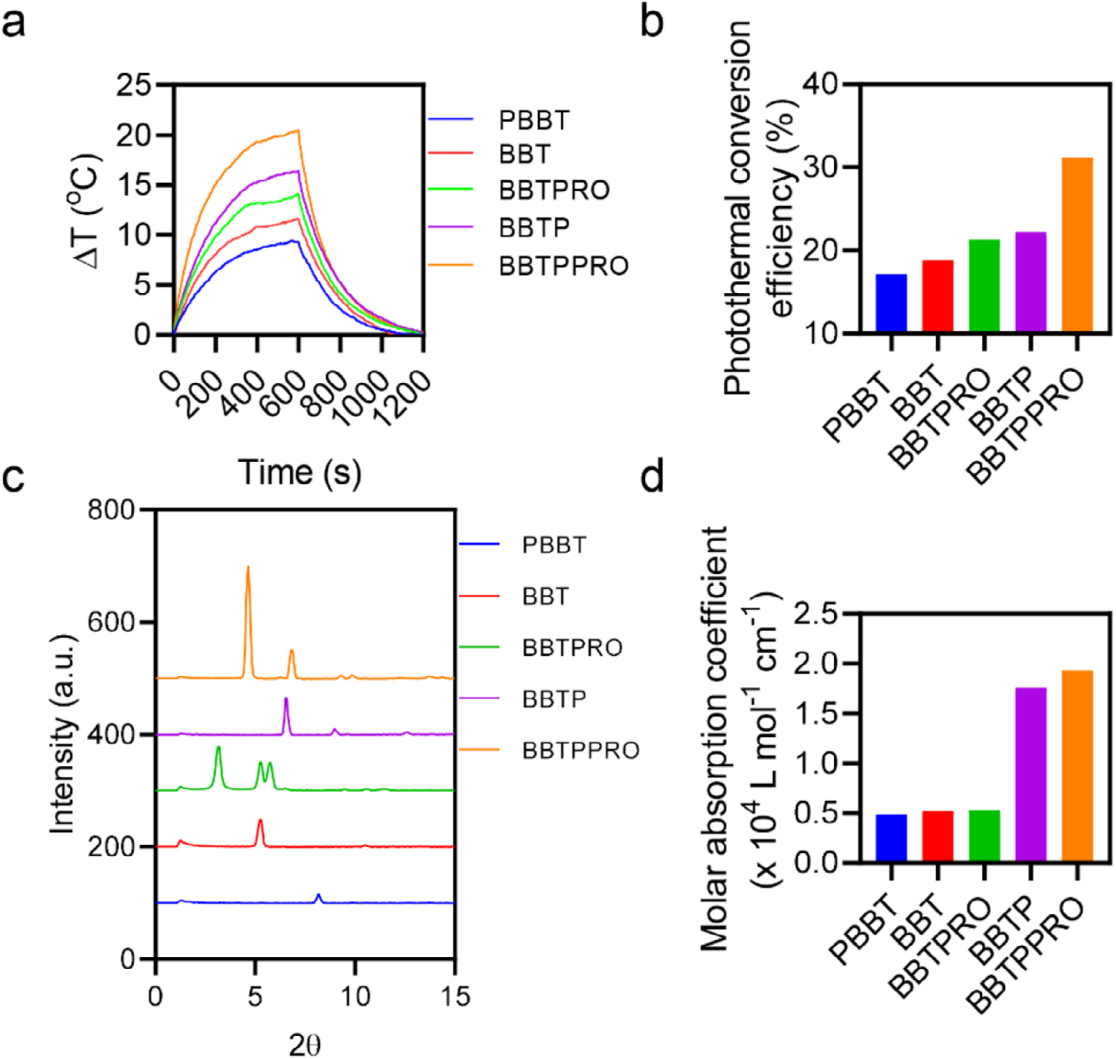
Photothermal conversion property of substrate‐free Raman molecules. a,b) Temperature elevations (ΔT) (a) and photothermal conversion efficiency (η) b) of the designed molecules (50 µm) in H_2_O/THF (95:5, v/v) under the laser irradiation (808 nm, 1 W cm^−2^) for 10 min, followed by another 10 min of cooling. c) GIWAXS spectra of various molecules. d) Molar absorption coefficient of molecules in THF at 808 nm.

We next evaluated the photothermal conversion properties of these substrate‐free Raman molecules by measurement of their temperature changes (**Figure** **3**a).^[^ ^29^, ^50^ ^]^ With the increase of side‐chain lengths and the introduction of phenyl groups, the photothermal conversion efficiency was enhanced (Figure 3b and Figure S21, Supporting Information). GIWAXS spectra showed that lamellar stacking (100) peak caused by alkyl side chains appeared at 2*θ* between 3° and 9° (Figure 3c).^[^ ^51^ ^]^ According to Bragg's law, stacking distance is negatively correlated with diffraction angle.^[^ ^52^ ^]^ The molecule BBTPRO showed remarkably increased molecular distance compared with PBBT (Figure 3c). Our data in line with the previous literature supported that long alkyl chains increased the intermolecular distance, beneficial to molecular motions and heat generation.^[^ ^36^, ^53^, ^54^ ^]^ Moreover, after modified with the phenyl groups, the absorption peaks of BBTP and BBTPPRO in THF were red shifted, resulting in significant increase in their molar absorption coefficients at 808 nm (Figure 3d). These results evidenced that the increase in the length of alkyl chains and the addition of electron donor, i.e., phenyl groups corporately contributed to the highest photothermal conversion efficiency of BBTPPRO (Figure 3b).

### 2.4 Raman Intensities and Photothermal Properties of BBTPPRO NPs

BBTPPRO with the highest Raman signal intensity and photothermal property was used for the preparation of micellular formulation BBTPPRO NPs. In the BBTPPRO NPs with the molar ratio of BBTPPRO to DSPE‐PEG to be 1.5 (i.e., 1:0.67), the hydrophobic BBTPPRO molecules were embedded in the core of micelles. The Raman intensity at peak 894 cm^−1^ of BBTPPRO NPs was close to that of BBTPPRO in the aggregates in the H_2_O/THF (95: 5, v/v) mixture (Figure S22, Supporting Information). To understand if BBTPPRO was self‐staked or interacted with the DSPE moiety of DSPE‐PEG, we measured the Raman scattering cross‐sections of BBTPPRO to be 3.74 × 10^−22^ cm^2^ per molecules in BBTPPRO NPs. This value was close to that of BBTPPRO in the aggregates in in the H_2_O/THF (95: 5, v/v) mixture (3.94 × 10^−22^ cm^2^ per molecule). This result indicated that the interaction between the DSPE moiety of DSPE‐PEG and BBTPPRO did not affect the self‐stacking of BBTPPRO in the core of micelles for enhanced Raman scattering.

Furthermore, we measured the Raman spectra of BBTPPRO NPs at various BBTPPRO/DSPE‐PEG (mol/mol) ratios. The Raman signal intensity was decreased with the decrease in the BBTPPRO/DSPE‐PEG ratio (Figure S23a, Supporting Information). When the BBTPPRO/DSPE‐PEG (mol/mol) ratios were decreased to 0.1 (i.e., 1:10), the S/N at 894 cm^−1^ decreased to 2.18, which was below 3 (Figure S23b, Supporting Information). This result indicated that the self‐stacking of BBTPPRO was completely disrupted by the interaction of DSPE‐PEG when tenfold amount of the surfactant was added. Collectively, the interaction between the BBTPPRO and the DSPE moiety affected the self‐stacking of BBTPPRO in the micelle in a ratio‐dependent manner. However, in the BBTPPRO NPs with the molar ratio of BBTPPRO to DSPE‐PEG to be 1.5, the BBTPPRO molecules were mainly self‐stacked with enhanced Raman scattering effect.

**Figure 4:**
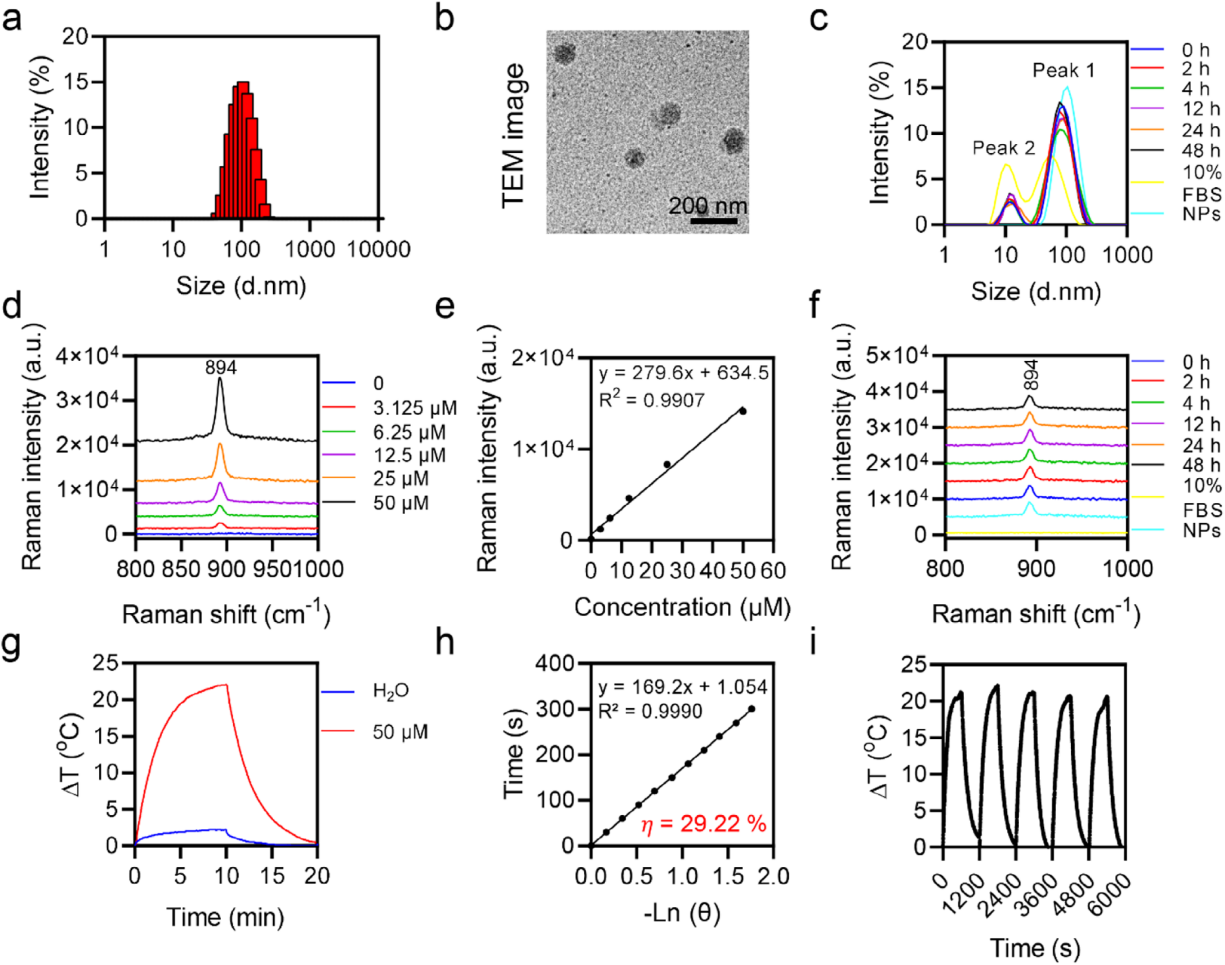
Raman and photothermal properties of BBTPPRO NPs. a) Size distribution of BBTPPRO NPs. b) Representative TEM image of BBTPPRO NPs. c) Size distribution of 10% FBS, BBTPPRO NPs in PBS, or BBTPPRO NPs in 10% FBS at different time points following mixture. Peak 1 from the nanoparticles; Peak 2 from FBS. d,e) Raman spectra (d) and intensity at 894 cm^−1^ (e) of BBTPPRO NPs at different concentrations excited at 785 nm, respectively. f) Raman spectra of 10% FBS, BBTPPRO NPs in PBS, or BBTPPRO NPs in 10% FBS at different time points following mixture. g) Temperature–time curves of H_2_O or BBTPPRO NPs (50 µm) in water under the laser irradiation (808 nm, 1 W cm^−2^) for 10 min, followed by another 10 min of cooling. h) Linear fitting of time from the cooling period versus the negative natural logarithm of driving force temperature of BBTPPRO NPs. i) Temperature elevations of BBTPPRO NPs (50 µm of BBTPPRO) during five cycles of heating‐cooling processes under the laser irradiation (808 nm, 1 W cm^−2^) for 10 min, followed by another 10 min of cooling.

The average particle size of BBTPPRO NPs with the molar ratio of BBTPPRO to DSPE‐PEG to be 1.5 was ≈105 nm by dynamic light scattering (DLS) analysis (**Figure** **4**a), consistent with the transmission electron micrograph (TEM) (Figure 4b). Compared with BBTPPRO in THF, the absorption peak of BBTPPRO NPs in water shifted from 785 to 800 nm (Figure S24, Supporting Information). BBTPPRO NPs were stable in 10% fetal bovine serum (FBS) with unchanged size distribution within 48 h (Figure 4c). The Raman signal of BBTPPRO NPs exhibited a linear correlation with their concentration (Figure 4d,e). BBTPPRO NPs did not show any changes in the Raman signal intensity after incubation with 10% FBS for 48 h or after laser irradiation for 10 min (Figure 4f and Figure S25). BBTPPRO NPs significantly elevated the solution temperature upon an 808 nm laser excitation (Figure 4g). The photothermal effect correlated with the concentration of BBTPPRO NPs (Figure S26, Supporting Information). The photothermal conversion efficiency of BBTPPRO NPs in aqueous solution at 808 nm was determined to be 29.22% (Figure 4h). During the five cycles of heating and cooling, the photothermal effect of BBTPPRO NPs remained unchanged, suggesting that BBTPPRO NPs were photostable (Figure 4i).

Indocyanine green (ICG) as the classic organic PTT agent, and Au nanorods as the relevant inorganic nanomaterials were used to conduct a comparison of photothermal properties with BBTPPRO NPs, respectively, since the absorption peaks of the above agents were all near 808 nm (green curve, Figure S24 and S27a, Supporting Information). The elevation of Au nanorod solution following laser irradiation (808 nm, 1 W cm^−2^) for 10 min was recorded to calculate the photothermal conversion efficiency to be 19.22%, lower than that of BBTPPRO NPs (29.22%) (Figure S27b,c, Supporting Information). After the laser exposure for 5.27 min, the temperature of ICG solution reached the peak then declined when the laser irradiation continued (Figure S27b, Supporting Information). This result was possibly attributed to photobleaching of ICG, which reduced the heating performance with a photothermal conversion efficiency of 18.10% (Figure S27d, Supporting Information). In summary, compared with ICG or Au nanorods, BBTPPRO NPs demonstrated superior photothermal conversion efficiency and photostability.

The cellular uptake of BBTPPRO NPs by CT26‐Luc mouse colon cancer cells was in concentration‐ and time‐dependent manners (Figure S28a, Supporting Information). The tumor cell‐killing effect following PTT was also dependent on the nanoparticle's concentration (Figure S28b, Supporting Information). BBTPPRO NPs did not show obvious dark cytotoxicity to mouse embryonic fibroblasts NIH 3T3 cells after incubation for 24 h at a concentration up to 400 µm (Figure S28c, Supporting Information). Blood chemistry or hematologic parameters remained unchanged in mice within one month after intravenous (i.v.) injection of BBTPPRO NPs at a dose of 20 mg kg^−1^ (Figure S29 and Table S1, Supporting Information). In addition, BBTPPRO NPs did not show noticeable toxicity in major organs analyzed by hematoxylin and eosin (H&E) staining (Figure S30, Supporting Information). These results demonstrated good biocompatibility of BBTPPRO NPs.

### 2.5 Comparison of Optical Physical Properties of SICTERS‐ and SERS‐Active Probes

**Figure 5:**
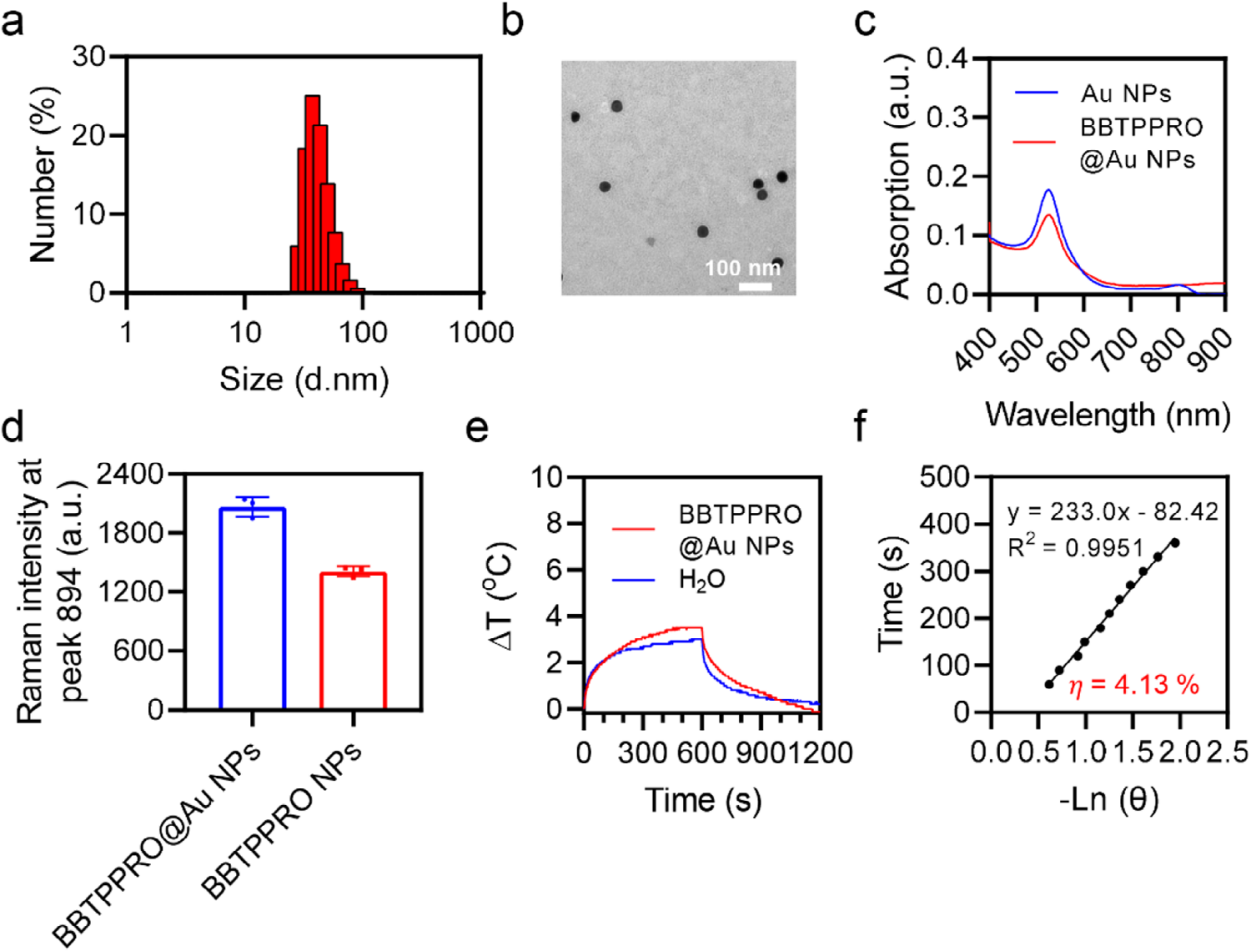
Characterization of Au NPs and BBTPPRO@Au NPs. a) Size distribution of Au NPs. b) Representative TEM image of Au NPs. c) UV–vis absorption spectra of Au NPs or BBTPPRO@Au NPs (0.01 mg mL^−1^ of Au) in H_2_O. d) Raman intensity at 894 cm^−1^ of BBTPPRO NPs (2.5 µm of BBTPPRO) or BBTPPRO@Au NPs (2.5 µm of BBTPPRO) in H_2_O excited at 785 nm. e) Temperature‐time curves of H_2_O or BBTPPRO@Au NPs (0.01 mg mL^−1^ of Au) in water under the laser irradiation (808 nm, 1 W cm^−2^) for initial 10 min, followed by another 10 min of cooling. f) Linear fitting of time from the cooling period versus the negative natural logarithm of driving force temperature for BBTPPRO@Au NPs.

To compare Raman scattering intensity by STCTERS with that by SERS, BBTPPRO was coated on the surface of Au nanoparticles (Au NPs) to prepare SERS‐active BBTPPRO@Au NPs. The average particle size of BBTPPRO@Au NPs was ≈40 nm by DLS analysis (**Figure** **5**a), consistent with the result of TEM (Figure 5b). The absorption spectra of Au NPs remained unchanged before and after the BBTPPRO coating (Figure 5c). At the same concentration of BBTPPRO (2.5 µm), the Raman intensity of BBTPPRO NPs (1408 a.u.) was close to that of BBTPPRO@Au NPs (2066 a.u.) (Figure 5d), suggesting that the Raman enhancement effect of SICTERS on each BBTPPRO molecule was similar to that of SERS. However, the BBTPPRO loading in BBTPPRO NPs (7.37 × 10^5^ per particle) was significantly higher than that in BBTPPRO@Au NPs (792 per particle). As a result, the Raman intensity of per particle in SICTERS‐active BBTPPRO NPs was 634‐fold higher than that in SERS‐active BBTPPRO@Au NPs. Meanwhile, the photothermal conversion efficiency of BBTPPRO NPs was 29.22%, which was sevenfold higher than that of BBTPPRO@Au NPs (4.13%) (Figure 5e,f).

### 2.6 Intraoperative Raman Image‐Guided PTT Using BBTPPRO NPs

To measure the maximum penetration depth of SICTERS Raman imaging in biological tissues, we sectioned the porcine skin into slices with the thickness of ≈0.4 mm each slice. Then, the BBTPPRO NPs solution (1 mg mL^−1^) in the glass capillary tube was covered with 1, 2, 3 and 4 pieces of porcine skin slices with skin tissue thicknesses of 0.4, 0.8, 1.2, and 1.6 mm, respectively, (Figure S31a, Supporting Information). The SICTERS‐based Raman imaging showed the ratio of Raman peak intensity (894 cm^−1^) to noise was above 3 when the sample was covered with porcine skin slices of 1.2 mm or less in depth (Figure S31b–e, Supporting Information). Under the intraoperative conditions without skin covering, such Raman imaging depth was sufficient for detection of tumors, especially microtumors in the colon.

**Figure 6:**
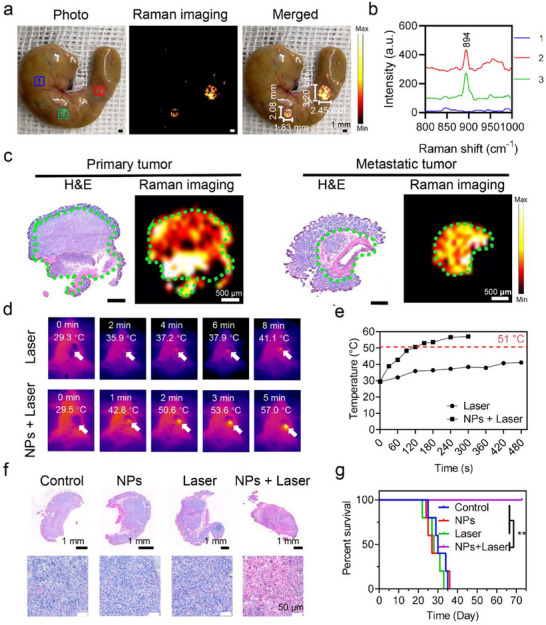
Intraoperative Raman image‐guided PTT of orthotopic CT26‐Luc mouse colon tumor by BBTPPRO NPs. a) Intraoperative Raman imaging (894 cm^−1^) of primary and metastatic tumor lesions of mice following i.v. injection of BBTPPRO NPs (7.5 mg kg^−1^). Region 1, cecum tissue. Region 2, primary tumor. Region 3, metastatic tumor. b) Raman spectra of regions in (a), respectively. c) Histological analysis with H&E staining and Raman imaging (894 cm^−1^) of primary and metastatic tumor sections, respectively. Green circles, tumor. d) Infrared thermal images of the tumor‐bearing mice irradiated with laser for different times. The mice were i.v. injected with BBTPPRO NPs (7.5 mg kg^−1^) or PBS at 24 h prior to the laser irradiation. In the NPs plus PTT group, colon tumor was identified by intraoperative Raman imaging and then treated with the laser irradiation (808 nm, 1 W cm^−2^). In the Laser group, primary tumor was visualized by eyes intraoperatively. Arrows, tumor sites. e) Temperature of the irradiated area‐time curves from the IR thermal imaging. f) Photographs of H&E staining of tumor of mice at 24 h following different treatments in (d). g) The survival curves of tumor‐bearing mice after different treatments. NPs, mice injected with BBTPPRO NPs (7.5 mg kg^−1^). Laser, 808 nm, 1 W cm^−2^. Control, mice injected with PBS alone. Statistical significance was calculated via the log‐rank Mantel–Cox test. ^**^ *p *<0.01, compared with NPs + Laser group.

Biodistribution results of BBTPPRO NPs in mice bearing orthotopic CT26‐Luc colon tumor confirmed that a higher accumulation in tumor tissues was achieved after 24 h compared with 1 h post‐injection (Figure S32, Supporting Information). Therefore, we performed intraoperative Raman image‐guided PTT after i.v. injection of BBTPPRO NPs (7.5 mg kg^−1^ of BBTPPRO) for 24 h. The intraoperative Raman imaging depicted the primary tumor with dimensions of 3.20 mm × 2.45 mm and the metastatic lesion with size of 2.08 mm × 1.83 mm, respectively, (**Figure** **6**a). The Raman signal at primary or metastatic tumor was clearly distinguished from that in the normal tissue (Figure 6b). Histological analysis with H&E staining confirmed the co‐localization of Raman signals in primary and metastatic tumor regions, respectively (Figure 6c). The intraoperative Raman image of a repeated mouse delineated a primary tumor with smaller size of 2.58 mm × 1.74 mm (Figure S33, Supporting Information). The tumor of mice injected with PBS did not have the Raman signals at 894 cm^−1^, in support of the specificity of Raman imaging by BBTPPRO NPs (Figure S34, Supporting Information). By contrast, after i.v. injection of SERS‐active BBTPPRO@Au NPs (7.5 mg kg^−1^ of Au), the tumor of mice did not have any Raman signals at 894 cm^−1^ (Figure S35, Supporting Information).

BBT NPs were prepared with the similar particle size as BBTPPRO NPs, with an average particle size of ≈101 nm (Figure S36a,b, Supporting Information). After intravenous injection of BBT NPs or BBTPPRO NPs (10 mg kg^−1^) for 24 h, we performed intraoperative Raman imaging of the orthotopic CT26‐Luc mouse colon tumors. Both nanoparticles enabled delineation of tumor via the SICTERS imaging (left, Figure S36c,d, Supporting Information). A distinct and sharp Raman peak at 894 cm^−1^ was observed in the tumor region, while no signal was detected in the cecum tissue (right, Figure S36c,d, Supporting Information). The intensity at 894 cm^−1^ of BBTPPRO NPs group was 1.23‐fold of that of BBT NPs group (Figure S36e, Supporting Information). Whereas, the S/N at 894 cm^−1^ at the colon tumor site in mice treated with BBTPPRO NPs was 18.59, resulting 3.21‐fold of that treated with BBT NPs (S/N = 5.80) (Figure S36f, Supporting Information). The results from both in vivo and in vitro experiments demonstrated that the introduction of longer alkyl chains and benzene rings significantly enhanced both the intensity and S/N of the Raman signal.

To illustrate the in vivo photothermal effects of BBTPPRO NPs, we located the tumor areas of mice under the guidance of intraoperative Raman imaging and utilized an 808 nm laser (1 W cm^−2^) to irradiate the tumor. In the NPs plus laser group, the temperature of tumor was raised rapidly to 51 °C (Figure 6d,e). Maintenance of such high temperature for 100 s was sufficient for thermal ablation of tumor cells.^[^ ^29^, ^55^, ^56^ ^]^ The result of H&E staining confirmed a complete necrosis of tumor of mice receiving BBTPPRO NPs plus PTT, which was characterized by extensive cytoplasmic acidophilia, pyknosis, and karyolysis (Figure 6f). The intraoperative Raman image‐guided PTT of both primary and metastatic tumors resulted in a complete cure of mice (5 out of 5) without recurrence till the end of experiment, i.e., 70 days (Figure 6g and Figures S37–S39, Supporting Information). By contrast, the mice injected with BBTPPRO NPs alone or treated with laser alone did not show prolonged survival times compared with the control (Figure 6g).

## 3 Discussion

The BPBBT molecule consists of a benzobisthiadiazole unit as the electron acceptor and triphenylamine units as the electron donors. The triphenylamine induces a twisted conformation in the molecular skeleton with the dihedral angle between the benzobisthiadiazole core and phenyl group of 35° based on DFT calculation. Furthermore, the rotation of the twisted BPBBT molecules is restricted in the aggregated state in the mixture of H_2_O/THF (95:5, v/v), thereby enhancing the fluorescence intensity through aggregation‐induced emission (AIE) behavior.^[^ ^29^ ^]^ By contrast, with the increase of H_2_O/THF ratio of the mixture, the fluorescence of all the designed molecules in this study was reduced and even quenched, suggesting an aggregation‐caused quenching (ACQ) effect (Figure S10, Supporting Information). The DFT calculation demonstrated that all the molecules in this study were plane‐structured with the dihedral angles between the benzobisthiadiazole core and the thiophene blocks less than 5° (Figure 1a). The strong intermolecular interactions in aggregate state caused by planar backbone led to ACQ behavior instead of AIE.

The Raman cross‐section of previously reported Raman tags such as alkynes (EdU), alkyne polymers (Carbow 2‐yne and Carbow 6‐yne), MARS dyes (MARS2228) and azo compounds (*p*‐Nitroazobenzene) is 2.41 × 10^−28^, 7.24 × 10^−27^, 2.37 × 10^−25^, 9.20 × 10^−26^ cm^2^ per molecule and ≈1–10 × 10^−27^ cm^2^, respectively.^[^ ^57^, ^58^ ^]^ The Raman scattering cross‐section of BBT per molecule in the BBT aggregates in the H_2_O/THF (95: 5, v/v) mixture under 785 nm excitation was calculated to be 2.59 × 10^−22^ cm^2^ per molecule according to the calculation method in our previous study.^[^ ^22^ ^]^ Using BBT as a reference, the Raman scattering cross‐sections of molecules PBBT, BBTPRO, BBTP, and BBTPPRO in the aggregates in the H_2_O/THF (95: 5, v/v) mixture were calculated to be 1.65 × 10^−22^, 2.95 × 10^−22^, 3.08 × 10^−22^, and 3.94 × 10^−22^ cm^2^ per molecule, respectively. And the Raman scattering cross‐section of SICTERS molecules is 10^2^–10^6^‐fold higher than that of these listed molecules.

DSPE‐PEG_2000_ was used to encapsulate BBTPPRO molecules to form micellar formulation BBTPPRO NPs. Due to the absence of targeting moieties on the micelle surface, BBTPPRO NPs lack the capability of active tumor targeting. Nanoparticles such as micelles with PEGylation can enhance their tumor penetration via the enhanced permeability and retention (EPR) effect.^[^ ^59^, ^60^ ^]^ Therefore, it was speculated that the accumulation of BBTPPRO NPs in the tumor was attributed to the EPR effect.

## 4 Conclusion

In summary, we unraveled the relationship between the molecular structures of the substrate‐free Raman molecules and their SICTERS and photothermal conversion properties by alkyl side chain and D–A engineering. The increase in the length of alkyl side chain and the introduction of phenyl groups increased molecular volume and lowered HOMO–LUMO energy gap, resulting in the enhanced SICTERS intensity and S/N of Raman signal. Moreover, this caused the extension of intermolecular distance and red‐shifted absorption, contributing to the elevated photothermal conversion effect. The optimized SICTERS‐active BBTPPRO NPs achieved the Raman intensity of per particle by 634‐fold and the photothermal conversion efficiency by sevenfold compared with SERS‐active BBTPPRO@Au NPs, respectively. Our findings demonstrated the principle of molecular design of substrate‐free Raman small molecules tuning both SICTERS and photothermal properties with theranostic functions.

## 5 Experimental Section

### Characterization of Compounds

Nuclear magnetic resonance (NMR) spectra were measured on a Bruker AV 400 spectrometer or a Bruker AV 600 spectrometer. Chemical shifts (δ) were reported in parts per million (ppm). ^1^H NMR spectra were recorded at 400 or 600 MHz in CDCl_3_.^13^C NMR spectra were recorded at 151 MHz and referenced to corresponding solvent resonance. Matrix‐assisted laser desorption/ionization time of flight mass spectrometry (MALDI‐TOF MS) spectra were recorded in positive reflection mode on a 5800 proteomic analyzer using an Nd: YAG laser (Applied Biosystems, USA). The geometry optimization was calculated at the level of B3LYP/6‐31G (d) using density functional theory (DFT) method with the Gaussian 09 program package. UV–vis–NIR absorption spectra were recorded on a UV‐2401 PC UV/vis spectrophotometer (Shimadzu, Japan). The NIR fluorescence emission spectra were measured using a fluorescence spectrometer (PTI QM40, USA). Raman spectra were collected using an inVia Raman microscope equipped with a 1040 × 256 pixels charge‐coupled device detector (Renishaw, UK). Grazing‐incidence wide‐angle X‐ray scattering (GIWAXS) spectra were collected using a small/wide‐angle X‐ray Scattering system (Xenocs, France).

### Density Functional Theory Calculations

All the S_0_ geometries of molecules were calculated using the Gaussian 09 software package with DFT at the B3LYP/6‐31G (d) level based on solvation of water, in which the polarizable continuum model (PCM) was employed to consider the effects of the solvents. The orbitals, energy levels, dipole moments, and dihedral angles of the molecules were obtained at the B3LYP/6‐31G (d) level with DFT calculations based on its optimized geometries. Van der Waals volume and polarizability of PBBT, BBT, BBTPRO, BBTP, and BBTPPRO were calculated using Multiwfn software.^[^ ^61^ ^]^

### Calculation of the Photothermal Conversion Efficiency

The photothermal conversion efficiency (*η*) is expressed as follows:^[^ ^29^, ^50^ ^]^

(1) $$\eta =\frac{hS\left(T_{\mathrm{Max}}-T_{S}\right)-Q_{C}}{I\left(1\;-10^{-A_{808}}\right)}$$

where *h* is heat transfer coefficient. *S* is the surface area of the container. The *Q_C_* represents heat dissipated from light absorbed by the quartz sample cell itself, using a quartz cuvette cell containing pure water. *T_Max_* is the maximum system temperature. *T_S_* is ambient temperature of the surroundings. *I* is 1.0 W cm^−2^. *A_808_* is the absorbance of compound in in H_2_O/THF (95:5, v/v) at 808 nm.

### Preparation and Characterization of BBTPPRO Nanoparticles (BBTPPRO NPs)

THF (800 µL) containing 0.5 mg of BBTPPRO and 1 mg of DSPE‐mPEG_2000_ was dripped into stirring water with tenfold volume. After THF evaporation by stirring the mixture in fume hood for 3 h, the resulting BBTPPRO NPs were obtained by filtration through a 0.22‐µm filter. The morphology of BBTPPRO NPs was observed by transmission electron micrograph (TEM, FEI Tecnai G2 20 TWIN). For the stability test, BBTPPRO NPs were added to 10% (v/v) fetal bovine serum (FBS, Meilunbio, Dalian, China) at room temperature to give a final concentration of 0.1 mg mL^−1^. The size distribution of the solution at different time points was measured by DLS (Malvern 3000, UK). Raman spectra were measured at the same time.

### Quantification of BBTPPRO in Nanoparticles

To measure the concentration of BBTPPRO, the molecule was extracted from each kind of nanoparticles using toluene under ultrasonication. The BBTPPRO concentration in toluene was calculated by measuring its absorbance at 780 nm with a standard curve.

### Cells and Animals

Mouse colon cancer CT‐26 cell line stably expressing luciferase CT26‐Luc (CT26.WT‐Fluc‐Neo) was obtained from Imanis Life Sciences (Rochester, MN, USA). Cells were cultured in RPMI 1640 supplemented with 10% FBS, penicillin (100 µg mL^−1^) and streptomycin (100 µg mL^−1^) at 37 °C and 5% CO_2_. BALB/c mice (male, 6–8 weeks, 20–22 g) and ICR mice (male, 6–8 weeks, 20–22 g) were ordered from Shanghai Lingchang Biological Co., Ltd. (Shanghai, China). All animals were allowed to adapt to the environment for at least one week before experiments, and housed under specific pathogen free conditions with free access to food and water. All animal experiments were performed under the guidance of Institutional Animal Care and Use Committee (IACUC) of School of Pharmacy, Fudan University.

### Intraoperative Raman Imaging

At 4 days after the tumor cell inoculation, the mice were i.v. injected with BBTPPRO NPs in PBS containing 7.5 mg kg^−1^ of BBTPPRO. After 24 h, the injection, the mice received an abdominal skin incision under anesthesia. The cecum exposure was exposed for intraoperative Raman imaging with an inVia Raman microscope (Renishaw, UK). Raman scanning was performed in Streamline high‐speed acquisition mode with 785‐nm laser, 124.8 µm step size and 0.3 s exposure time. Characteristic peak at 894 cm^−1^ of BBTPPRO NPs was selected for the image processing. The Raman images were generated and analyzed by a signal to baseline algorithm (WiRE4.3 software, Renishaw, UK), with specific steps including cosmic removal, noise filtering, baseline correction, and smoothing according to instrument operating procedures.

### Intraoperative Raman Image‐Guided PTT

Four days after the tumor inoculation, the CT26‐Luc colon cancer‐bearing mice were randomly divided into four groups (*n =* 5). In the Control group, the mice received an i.v. injection of PBS and no other treatment. In the NPs group, the mice received an i.v. injection of BBTPPRO NPs (7.5 mg kg^−1^) without other treatment. In the Laser group, mice received an i.v. injection of PBS. After 24 h, only the primary tumor lesion was visualized with eye intraoperatively and treated with laser radiation (808 nm, 1 W cm^−2^, and 8 min). In the NPs + Laser group, the mice received an i.v. injection of BBTPPRO NPs (7.5 mg kg^−1^). After 24 h, both primary and metastatic tumors were visualized under the intraoperative Raman imaging followed by PTT (primary tumor, 1 W cm^−2^ and 5 min; metastatic tumor, 1 W cm^−2^, 8 min and 808 nm). The cecum was repositioned to the abdominal cavity following the PTT. The skin was closed with suture.

### Statistical analysis

Statistical analysis was performed using the GraphPad Prism 8 software. Data were presented as the mean ± SD. Statistical significance was determined by unpaired *t*‐tests, one‐way or two‐way analysis of variance (ANOVA) with Tukey's multiple comparisons test. Survival was analyzed using the Kaplan–Meier survival curves. The curves were compared with the log‐rank Mantel‐Cox test (^*^ *p* <0.05, ^**^ *p* <0.01, ^***^ *p* <0.001, ^****^ *p* <0.0001).

## Conflict of Interest

The authors declare the following competing financial interest(s): W.L. and S.Y. submitted a patent application related to the findings in the manuscript.

## Supporting information



Supporting Information

Supplemental Movie1

Supplemental Movie2

Supplemental Movie3

Supplemental Movie4

Supplemental Movie5

## Data Availability

The data that support the findings of this study are available from the corresponding author upon reasonable request.
