# Supplementary material for: Engineered Substrate‐Free Small Molecules for Enhanced Raman Scattering and Photothermal Conversion Efficiency
Source: Adv Sci (Weinh). 2025 Sep 5;12(44):e05467. doi: 10.1002/advs.202505467 (PMC12667519; doi:10.1002/advs.202505467)
Supplement: Supplementary file 1 — Supporting Information [file ADVS-12-e05467-s004.docx]

Supporting Information

Engineered Substrate-Free Small Molecules for Enhanced Raman Scattering and Photothermal Conversion Efficiency

Sheng Yu, Shuai Gao, Yongming Zhang, Sihang Zhang, Jingwen Sun, Wenxian Zhang, Tingting Li, Kai Cui, Zeyu Xiao,* and Wei Lu*

**Experimental section**

**Chemical synthesis.**

The synthesis procedures were shown in Figure S1.

*Synthesis of 4,7-bis(4-(2-ethylhexyl)thiophen-2-yl)benzo[1,2-c:4,5-c′]bis[1,2,5]thiadiazole (compound* ***6****, BBT)*. To a solution of Compound **3** (1.0 g, 5.10 mmol) in dry tetrahydrofuran (THF) at −78 °C was added n-BuLi (1.6 M, 4.78 mL) dropwise. After 30 min, a solution of SnBu_3_Cl in hexane (1.0 M, 8 mL) was added and the reaction mixture was warmed to ambient temperature for 12 h. After a complete conversion of the starting material, the reaction was quenched with water and extracted with CH_2_Cl_2_. The organic layers were dried over Na_2_SO_4_ and concentrated under reduced pressure. The crude product was obtained as orange oil which can be used in the next step without further purification.

A mixture of the crude product above and 4,7-dibromo-benzo [1,2-*c*:4,5-*c*′]bis([1,2,5]thiadiazole (compounds **1**, 0.78 g, 2.23 mmol) in 1,4-dioxane was bubbled with argon for 20 min. Pd[PPh_3_]_4_ (0.23 g, 0.20 mmol) was added to the above mixture. The mixture was heated at 105 °C for 12 h under argon atmosphere. Then, the solvent was removed in vacuo. The residue was chromatographed (petroleum ether / EtOAc = 120:1, v/v) on silica gel to get BBT as a blue solid (0.73 g, 56% yield). ^1^H NMR (400 MHz, CDCl_3_) δ 8.81 (s, 2H), 7.27 (s, 2H), 2.71 (dd, *J* = 6.2, 2.5 Hz, 4H), 1.72 (dt, *J* = 13.0, 3.8 Hz, 2H), 1.41 – 1.28 (m, 16H), 1.01 – 0.86 (m, 12H). ^13^C NMR (151 MHz, CDCl_3_) δ 150.55, 142.29, 136.50, 133.95, 126.50, 113.00, 39.79, 33.97, 31.90, 28.30, 25.07, 22.51, 13.60, 10.30. MALDI-TOF MS Calcd for: C_30_H_37_N_4_S_4_^-^ ([M−H]^−^): 582.2013. Found: 582.2315.

*Synthesis of 4,7-bis(4-(2-propyl)thiophen-2-yl)benzo[1,2-c:4,5-c′]bis[1,2,5]thiadiazole (compound* ***5****, PBBT).* Compound **5** was prepared in a manner similar to that of compound **6**. Yield: 46%; blue solids. ^1^H NMR (600 MHz, CDCl_3_) δ 8.84 (s, 2H), 7.32 (d, *J* = 12.0 Hz, 2H), 2.76 (t, *J* = 7.6 Hz, 4H), 1.80 (dq, *J* = 14.8, 7.4 Hz, 4H), 1.05 (t, *J* = 7.3 Hz, 6H). ^13^C NMR (151 MHz, CDCl_3_) δ 151.05, 144.09, 137.43, 134.12, 126.49, 113.72, 32.81, 23.93, 14.13. MALDI-TOF MS Calcd for: C_20_H_17_N_4_S_4_^-^ ([M−H]^−^): 442.0409. Found: 442.1567.

*Synthesis of 4,7-bis(4-(2-**hexyldecyl)thiophen-2-yl)benzo[1,2-c:4,5-c′]bis[1,2,5]thiadiazole (compound* ***7****, BBTPRO).* Compound **7** was prepared in a manner similar to that of compound **6**. Yield: 40%; blue solids. ^1^H NMR (600 MHz, CDCl_3_) δ 8.78 (s, 2H), 7.26 (d, *J* = 1.8 Hz, 2H), 2.71 (d, *J* = 6.8 Hz, 4H), 1.82 – 1.71 (m, 2H), 1.39 – 1.34 (m, 12H), 1.33 – 1.23 (m, 36H), 0.91 – 0.85 (m, 12H). ^13^C NMR (151 MHz, CDCl_3_) ) δ 150.49, 142.22, 136.47, 134.01, 126.52, 112.95, 38.38, 34.32, 32.78, 32.77, 31.34, 31.31, 29.49, 29.16, 29.08, 28.78, 26.07, 26.04, 22.11, 22.07, 13.52, 13.50. MALDI-TOF MS Calcd for: C_46_H_69_N_4_S_4_^-^ ([M−H]^−^): 806.4478. Found: 806.5529.

*Synthesis of* *4,7-bis(5-bromo-(4-(2-ethylhexyl)thiophen-2-yl)benzo[1,2-c:4,5-c′]bis[1,2,5]thiadiazole (compound* ***8****, Br-BBT).* To a solution of BBT (0.20 g, 0.34 mmol) in chloroform at 0 °C was added N-bromosuccinimide (NBS, 0.125 g, 0.70 mmol) in dry N,N-dimethylformamide (DMF) dropwise. After stirring for 5 min, the reaction was quenched with ice water and extracted with CH_2_Cl_2_. The organic layers were washed with saturated aqueous brine before being dried over Na_2_SO_4_. After the evaporation of solvents, the residue was purified by column chromatography on silica gel with petroleum ether: EtOAc (150:1, v/v) as the eluent to afford compound **8** (192 mg, 76 % yield) as a blue solid. ^1^H NMR (600 MHz, CDCl_3_) δ 8.55 (s, 2H), 2.63 (d, *J* = 7.3 Hz, 4H), 1.80 – 1.75 (m, 2H), 1.45 – 1.33 (m, 16H), 0.97 (t, *J* = 7.4 Hz, 6H), 0.92 (t, *J* = 7.0 Hz, 6H). ^13^C NMR (151 MHz, CDCl_3_) δ 149.89, 141.62, 136.38, 133.35, 117.31, 111.74, 39.39, 33.21, 31.91, 28.18, 25.16, 22.54, 13.60, 10.32. MALDI-TOF MS Calcd for: C_30_H_37_Br_2_N_4_S_4_^+^ ([M+H]^+^): 742.0146. Found: 742.0602.

*Synthesis of 4,7-bis(5-bromo-(4-(2-**hexyldecyl)thiophen-2-yl)benzo[1,2-c:4,5-c′]bis[1,2,5]thiadiazole (compound* ***9****, Br-BBTPRO).* Compound **9** was prepared in a manner similar to that of compound **8**. Yield: 80%; blue solids. ^1^H NMR (600 MHz, CDCl_3_) δ 8.39 (s, 2H), 2.59 (d, *J* = 7.2 Hz, 4H), 1.81 (d, *J* = 5.2 Hz, 2H), 1.40 – 1.22 (m, 48H), 0.92 – 0.84 (m, 12H). ^13^C NMR (151 MHz, CDCl_3_) δ 150.08, 142.03, 136.80, 133.83, 117.93, 111.92, 38.53, 34.03, 33.37, 33.35, 31.94, 31.89, 30.12, 29.78, 29.68, 29.38, 26.55, 26.51, 22.71, 22.64, 14.11, 14.06. MALDI-TOF MS Calcd for: C_46_H_69_Br_2_N_4_S_4_^+^ ([M+H]^+^): 964.2672. Found: 964.1844.

*Synthesis of 4,7-bis(4-(2-ethylhexyl)-5-phenylthiophen-2-yl)benzo[1,2-c:4,5-c′]bis[1,2,5]thiadiazole (compound* ***11****, BBTP).* To a solution of compound **10** (66 mg, 0.54 mmol) and compound **8** (100 mg, 0.135 mmol) in 1,4-dioxane (10 mL) was bubbled with argon for 5 min. Then, 2 M K_2_CO_3_ (0.34 mL) and Pd[PPh_3_]_4_ (15.6 mg, 0.014 mmol) were added to the reaction mixture under an argon atmosphere. The reaction mixture was heated at 105 °C for 2 h and then concentrated in vacuo. The residue was dissolved in CH_2_Cl_2_, and washed with water, saturated aqueous brine and dried over Na2SO4. The combined organic layers were concentrated and the resulting product was purified using a column chromatography (petroleum ether: EtOAc = 150:1, v/v) to give the desired compound 11 as a green solid (74 mg, 75%). ^1^H NMR (400 MHz, CDCl_3_) δ 8.88 (s, 2H), 7.63 (d, *J* = 6.9 Hz, 4H), 7.49 (t, *J* = 7.8 Hz, 4H), 7.41 (t, *J* = 6.0 Hz, 2H), 2.80 (d, *J* = 5.9 Hz, 4H), 1.80 – 1.70 (m, 2H), 1.41 – 1.27 (m, 8H), 1.23 (d, *J* = 2.2 Hz, 8H), 0.89 – 0.79 (m, 12H). ^13^C NMR (151 MHz, CDCl_3_) δ 150.66, 144.12, 138.50, 135.38, 135.14, 134.08, 128.83, 127.88, 127.10, 112.61, 39.92, 32.15, 31.96, 28.04, 25.21, 22.45, 13.50, 10.18. MALDI-TOF MS Calcd for: C_42_H_47_N_4_S_4_^+^ ([M+H]^+^): 736.2708. Found: 736.1809.

*Synthesis of 4,7-bis(4-(2-hexyldecyl)-5-phenylthiophen-2-yl)benzo[1,2-c:4,5-c′]bis[1,2,5]thiadiazole (compound* ***12****,* *BBTPPRO).* Compound **12** was prepared in a manner similar to that of compound **11**. Yield: 70%; green solids. ^1^H NMR (400 MHz, CDCl_3_) δ 8.88 (s, 2H), 7.61 (d, *J* = 7.1 Hz, 4H), 7.46 (t, *J* = 7.3 Hz, 4H), 7.38 (t, *J* = 7.1 Hz, 2H), 2.78 (d, *J* = 6.9 Hz, 4H), 1.83 – 1.72 (m, 2H), 1.23 (d, *J* = 19.8 Hz, 48H), 0.85 (t, *J* = 6.4 Hz, 12H). ^13^C NMR (151 MHz, CDCl_3_) δ 150.67, 144.11, 138.74, 138.52, 135.47, 134.08, 128.82, 127.89, 127.10, 112.62, 38.47, 32.83, 32.80, 31.30, 29.43, 29.10, 29.03, 28.75, 25.81, 22.08, 22.05, 13.49. MALDI-TOF MS Calcd for: C_58_H_79_N_4_S_4_^+^ ([M+H]^+^): 958.5104. Found: 958.1652.

**Preparation and** **intraoperative Raman imaging of BBT nanoparticles (BBT NPs).**

THF (500 μL) containing 0.5 mg of BBT and 1 mg of DSPE-mPEG_2000_ was dripped into stirring water with 10-fold volume under ultrasonic conditions. After THF evaporation by stirring the mixture in fume hood for 4 h, the resulting BBT NPs were obtained by filtration through a 0.22-µm filter.

In intraoperative Raman imaging comparative test between BBT NPs and BBTPPRO NPs, the mice were i.v. injected with BBT NPs (10 mg/kg of BBT) or BBTPPRO NPs (10 mg/kg) in PBS At 4 days after the tumor cell inoculation. Twenty-four hours after the injection, the mice received an abdominal skin incision under anesthesia. The cecum exposure was exposed for intraoperative Raman imaging with an inVia Raman microscope (Renishaw, UK). Raman scanning was performed in Streamline high-speed acquisition mode with 785-nm laser, 124.8 μm step size and 0.3 s exposure time. Characteristic peak at 894 cm^−1^ of BBT NPs or BBTPPRO NPs was selected for the image processing. The Raman images were generated and analyzed by a signal to baseline algorithm (WiRE4.3 software, Renishaw, UK).

**Preparation and** **characterization of BBTPPRO@Au nanoparticles (BBTPPRO@Au NPs).**

To prepare the SERS-active BBTPPRO@Au NPs, the Au NPs with a diameter of ~40 nm was synthesized according to the previously reported method^[1]^. The synthesized Au NPs were washed three times by centrifuge at 11000 rpm for 5 min and then dispersed in 10 mL of cetyltrimethylammonium chloride (CTAC) aqueous solution (0.5 mM). The morphology of Au NPs was examined by High Contrast Transmission Electron Microscope (Hitachi HT7800, Japan). Then, the corresponding amount of BBTPPRO and DSPE-mPEG_2000_ (0.1 mg) in THF (0.1 mL) was added under vigorous sonication for 5 min. The final concentration of Au was 1.27 mM.

The number of BBTPPRO NPs and Au NPs were measured by Nanoparticle Tracking Analysis (NTA) (Particle Metrix, Meerbusch, Germany), respectively. The prepared BBTPPRO NPs and Au NPs were diluted to 10^7^−10^8^ particles/mL and injected into the sample tank using a syringe. The software (ZetaView 8.04.02 SP2) was used for data acquisition and analysis.

**Photothermal stability tests.**

The aqueous solutions of BBTPPRO NPs were irradiated with an 808 nm laser (1 W/cm^2^). Raman spectra of BBTPPRO NPs in aqueous solutions were measured at different time points. For anti-photobleaching study, the temperatures of the sample solutions were recorded during five cycles of heating and cooling processes. During each heating−cooling cycle, the samples were irradiated with the NIR laser for the first 10 min to reach to a steady state. Following removal of the laser, the samples were allowed to cool down naturally to the ambient temperature for another 10 min.

***In vitro* cytotoxicity.**

NIH 3T3 cells (5 × 10^3^ per well, the American Type Culture Collection, CRL-1658) were cultured in 96-well microplates for 24 h before the experiment. The cells were then incubated with a series of concentrations of BBTPPRO NPs for another 24 h. Subsequently, 20 µL of methyl thiazolyl tetrazolium (MTT) solution (5 mg/mL) was added to each well. After 2-h incubation, the supernatant was replaced with 150 µL of dimethyl sulfoxide (DMSO) to dissolve crystals. The absorbance was measured at 570 nm using a Bio-Rad 550 microplate reader (Bio-Tek, USA).

Cellular uptake of BBTPPRO NPs.

CT26-Luc cells were seeded into 12-well plates with the complete culture medium at a density of 3 × 10^5^ of cells per well for 24 h. The medium was replaced with the culture medium containing 50 μM, 100 μM or 200 μM of BBTPPRO without FBS. After 2 h or 4 h, the cells were washed three times with cold phosphate buffered saline (PBS) and digested by 1 × trypsin (0.25% of Trypsin-Ethylene Diamine Tetraacetic Acid (EDTA)). After ultrasonication, BBTPPRO was extracted using toluene followed by quantification with a fluorescence spectrometer (PTI QM40, USA) at 960 nm excited by 808 nm.

Photothermal therapy (PTT) experiment *in vitro***.**

CT26-Luc cells were seeded into 96-well plates and incubated with different concentrations of BBTPPRO NPs. After 4 h, the supernatant was removed and the cells were irradiated with or without an 808 nm laser at 1 W/cm^2^ for 5 min. After the laser irradiation, the cells were incubated with complete culture medium at 37 °C for another 12 h. Subsequently, 20 µL of MTT solution (5 mg/mL) was added to each well. After another 2-h incubation, the supernatant was replaced with 150 µL of DMSO to dissolve crystals. The absorbance was measured at 570 nm using a Bio-Rad 550 microplate reader. The cell viability of normal tumor cells without any treatment was set as 100%.

**Biodistribution of BBTPPRO in the tumor-bearing mice.**

BALB/c mice bearing orthotopic CT26-Luc colon tumor were established according to our previously reported method.^[2]^ At 4 days after the tumor cell inoculation, the tumor-bearing mice were i.v. injected with BBTPPRO NPs (20 mg/kg) and euthanized at 1 h or 24 h post-injection (*n =* 5). Major organs including heart, liver, spleen, lung, kidneys, brain, cecum, tumor and cecal contents were collected and weighted. A portion of liver or whole heart, spleen, lung, kidney, brain, cecum or cecal contents was mixed with 1 mL of PBS. Tumor was mixed with 400 µL of PBS. The mixture was homogenized by a homogenizer (Jinxin, China) for 60 s in an ice bath. To extract BBTPPRO from different samples, 500 µL of toluene was added into 100 µL of the tissue homogenate. The mixture was emulsified by ultrasonic cell crusher (Scientz, China) for 60 s under ice bath. After centrifugation at 15,000 rpm for 10 min, the supernatant was transferred to quartz cuvette cells for measurement of the fluorescence emission of BBTPPRO at 960 nm excited by 808 nm using a fluorescence spectrometer (PTI QM40, USA). The blank tissue samples were added with BBTPPRO NPs standard solution of different concentrations, and received the same treatment for the calibration and validation test.

**Histologic analysis.**

For tumor imaging, the primary and metastatic tumor were identified through the intraoperative Raman imaging, and were removed under the guidance of the imaging. Then, the primary and metastatic tumor were embedded in optimal cutting temperature compound (OCT, Sakura Finetek, Japan) for frozen sectioning. Raman imaging of the tissue sections with a thickness of 50 μm was acquired using the Renishaw Streamline function,785-nm laser, 87.4 μm step size and 0.3 s exposure time. Adjacent tissue sections with a thickness of 8 μm were used for hematoxylin and eosin (H&E) staining.

**Supplementary Figures**


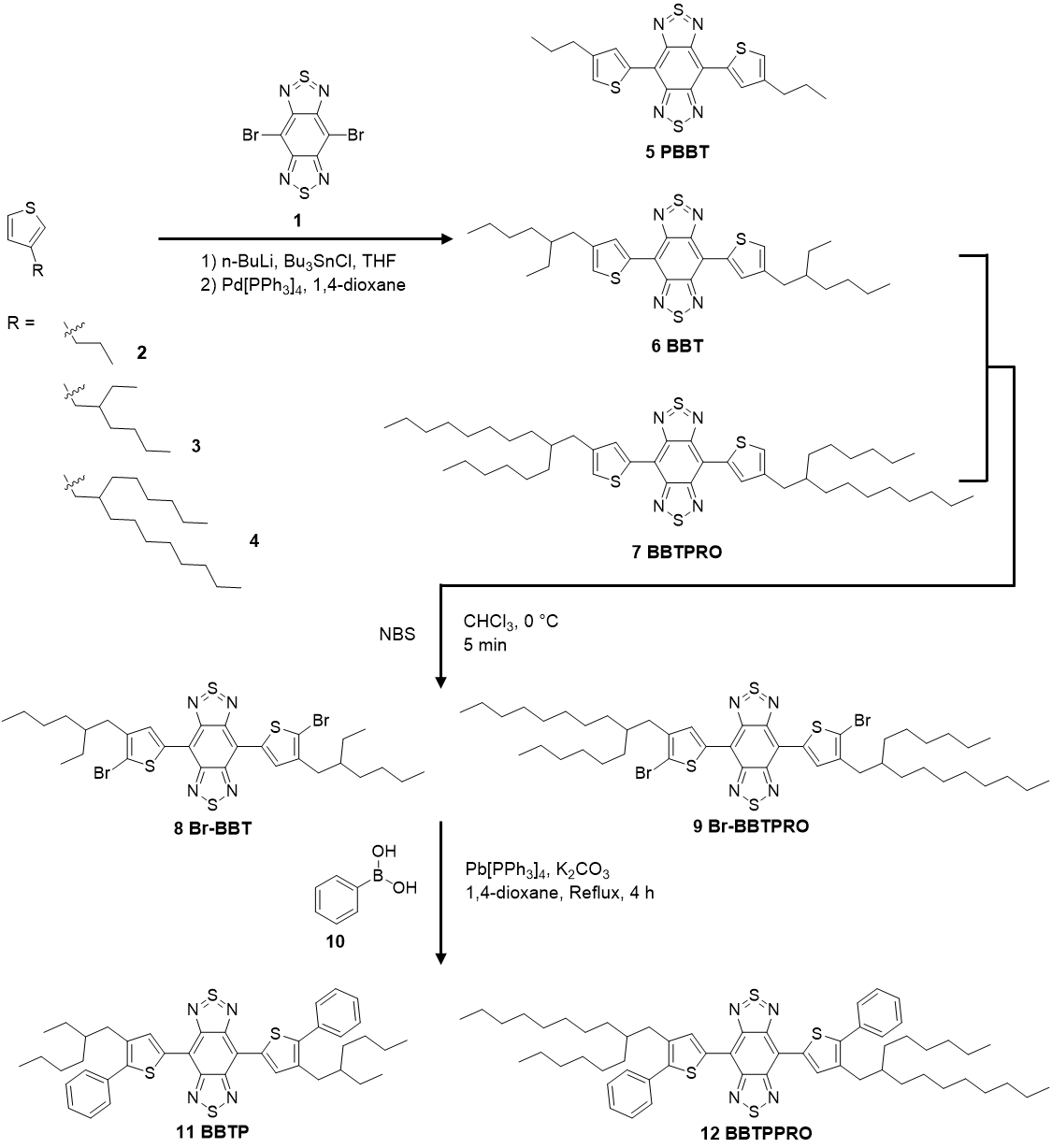


**Figure S1.** Synthesis route of substrate-free Raman molecules.


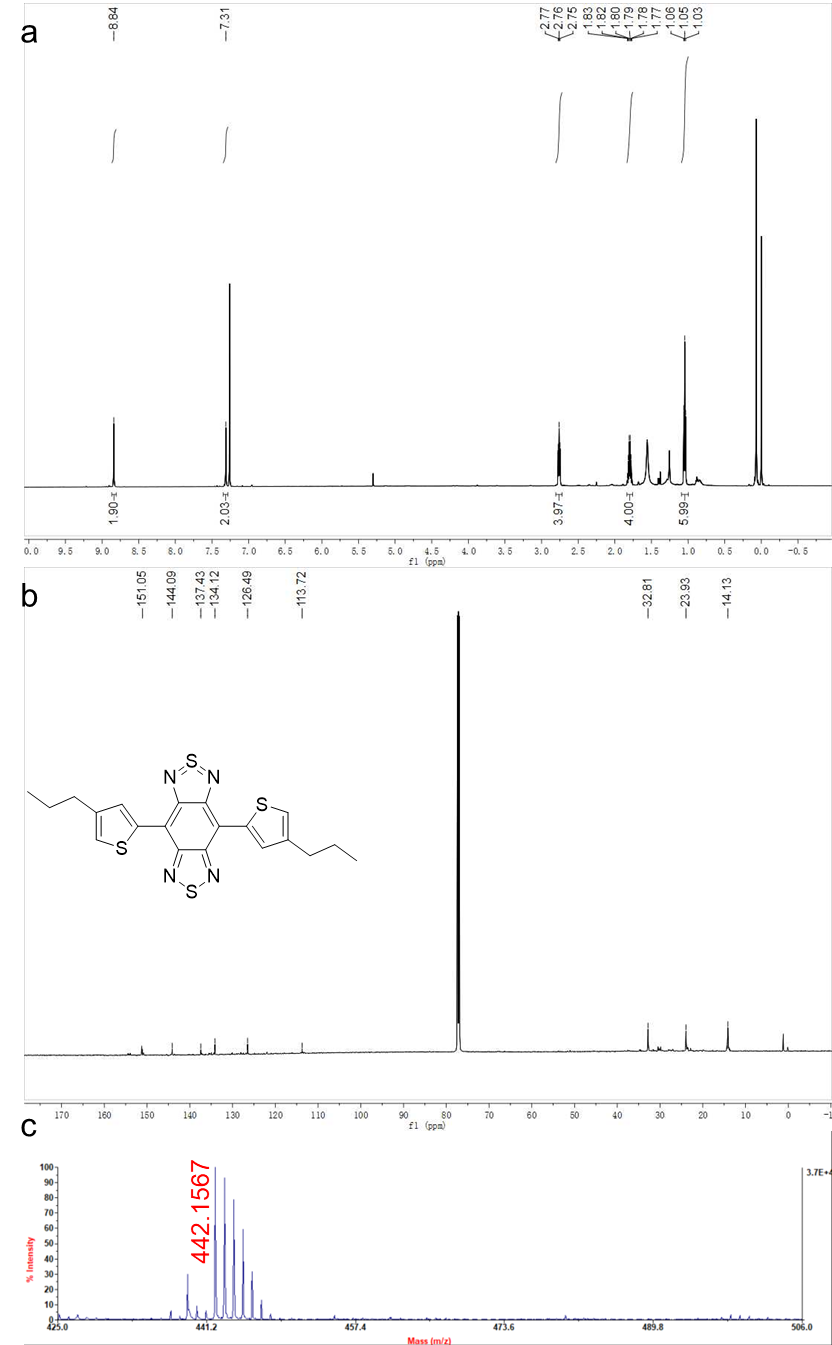


**Figure S2.** (a) ^1^H NMR spectra of PBBT in CDCl_3_. (b) ^13^C NMR spectra of PBBT in CDCl_3_. (c) MALDI-TOF (MS) spectrum of PBBT.


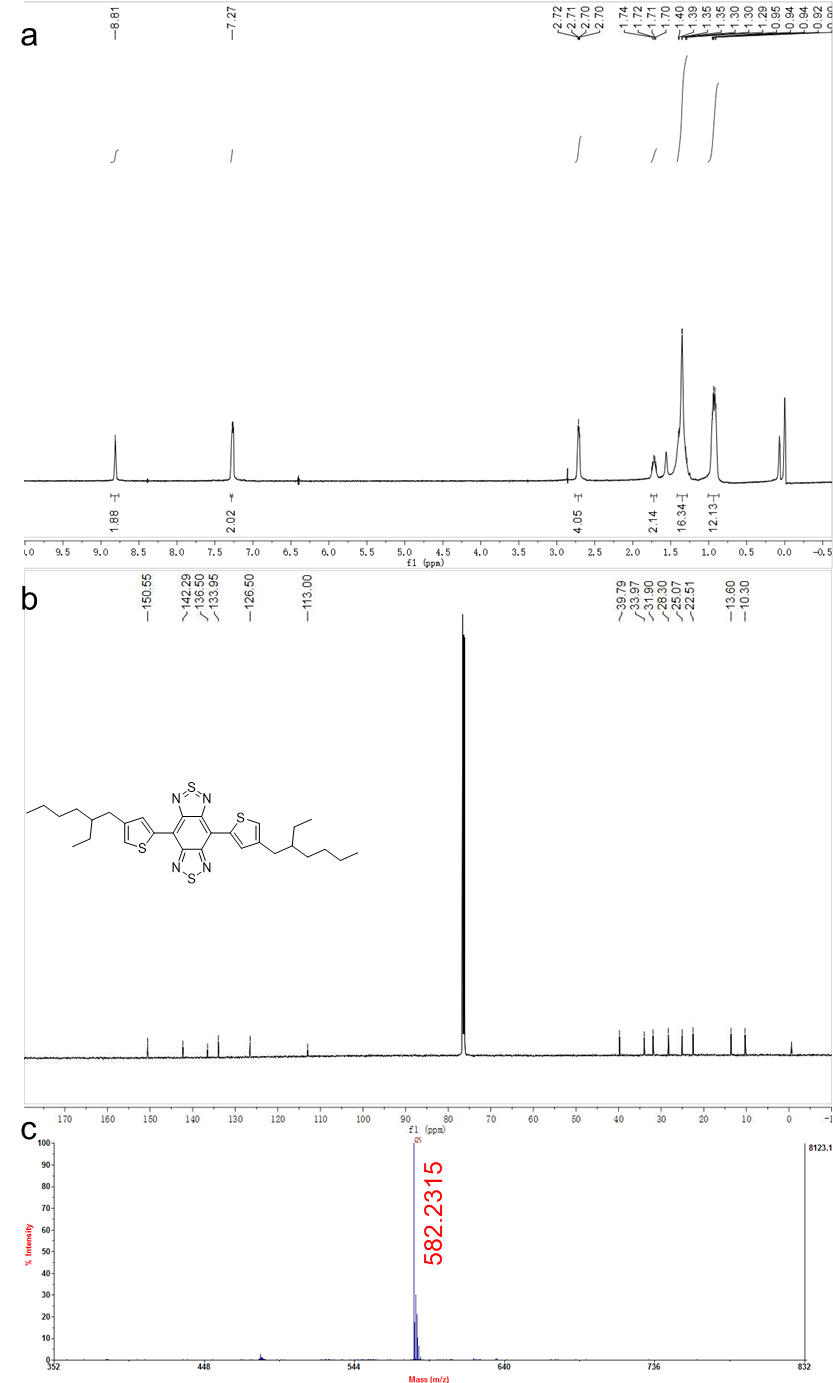


**Figure S3.** (a) ^1^H NMR spectra of BBT in CDCl_3_. (b) ^13^C NMR spectra of BBT in CDCl_3_. (c) MALDI-TOF (MS) spectrum of BBT.


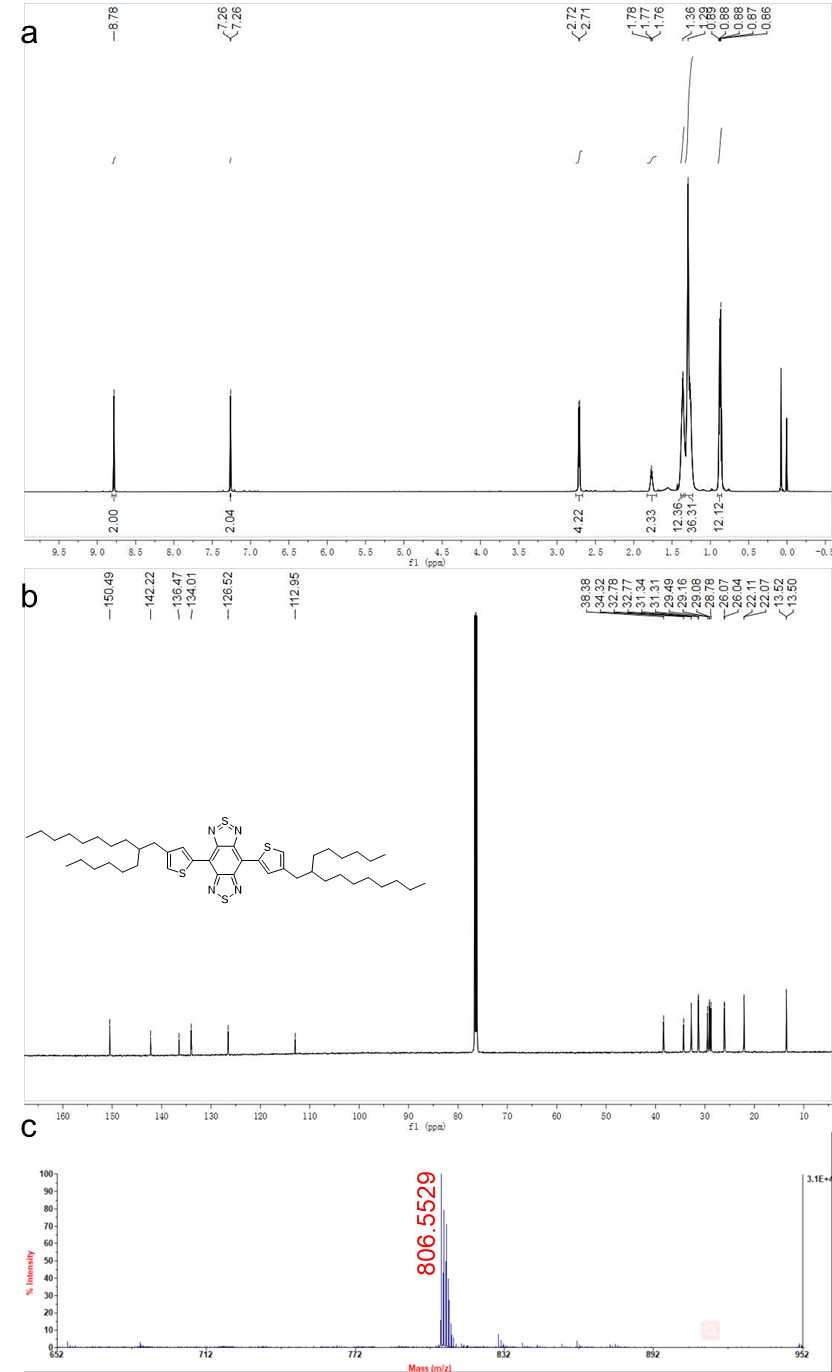


**Figure S4.** (a) ^1^H NMR spectra of BBTPRO in CDCl_3_. (b) ^13^C NMR spectra of BBTPRO in CDCl_3_. (c) MALDI-TOF (MS) spectrum of BBTPRO.


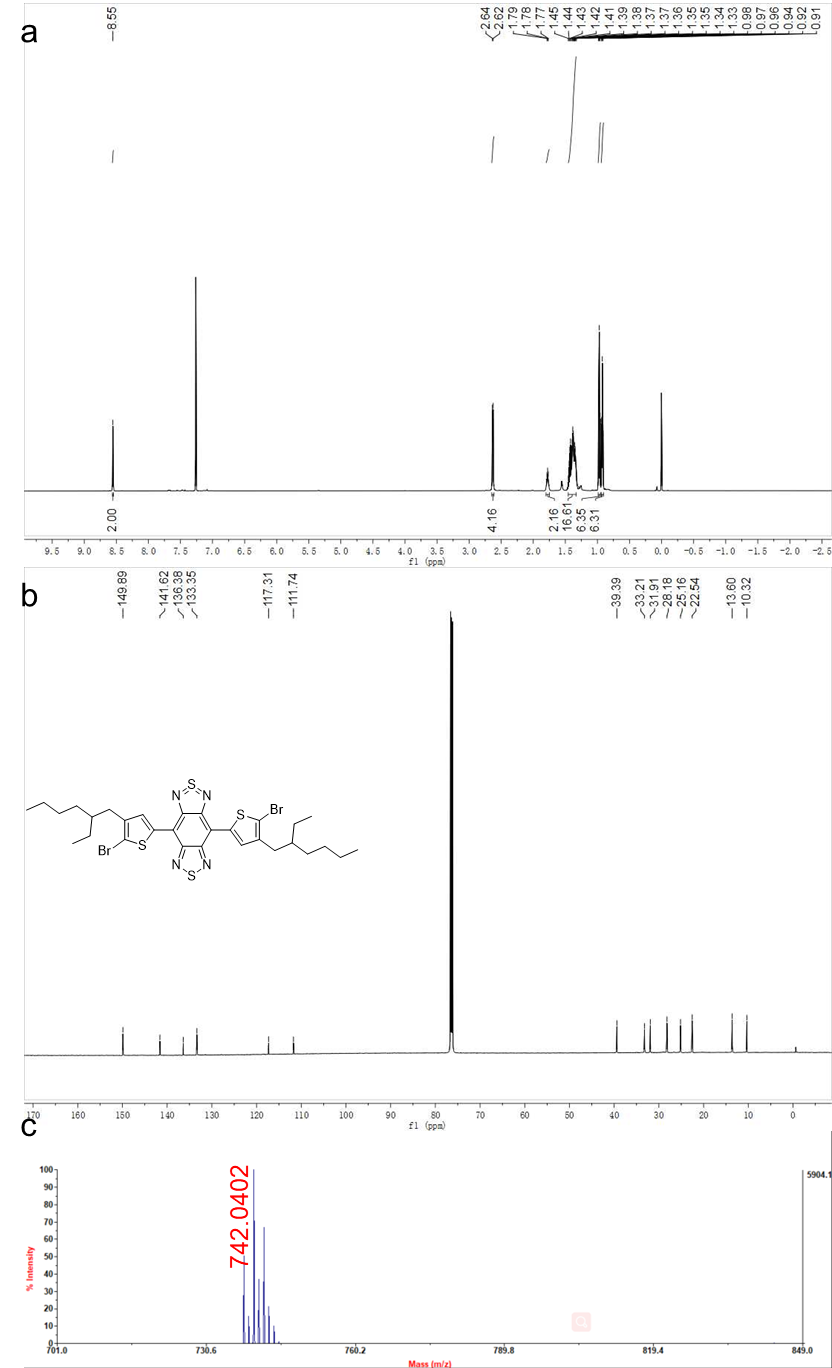


**Figure S5.** (a) ^1^H NMR spectra of Br-BBT in CDCl_3_. (b) ^13^C NMR spectra of Br-BBT in CDCl_3_. (c) MALDI-TOF (MS) spectrum of Br-BBT.


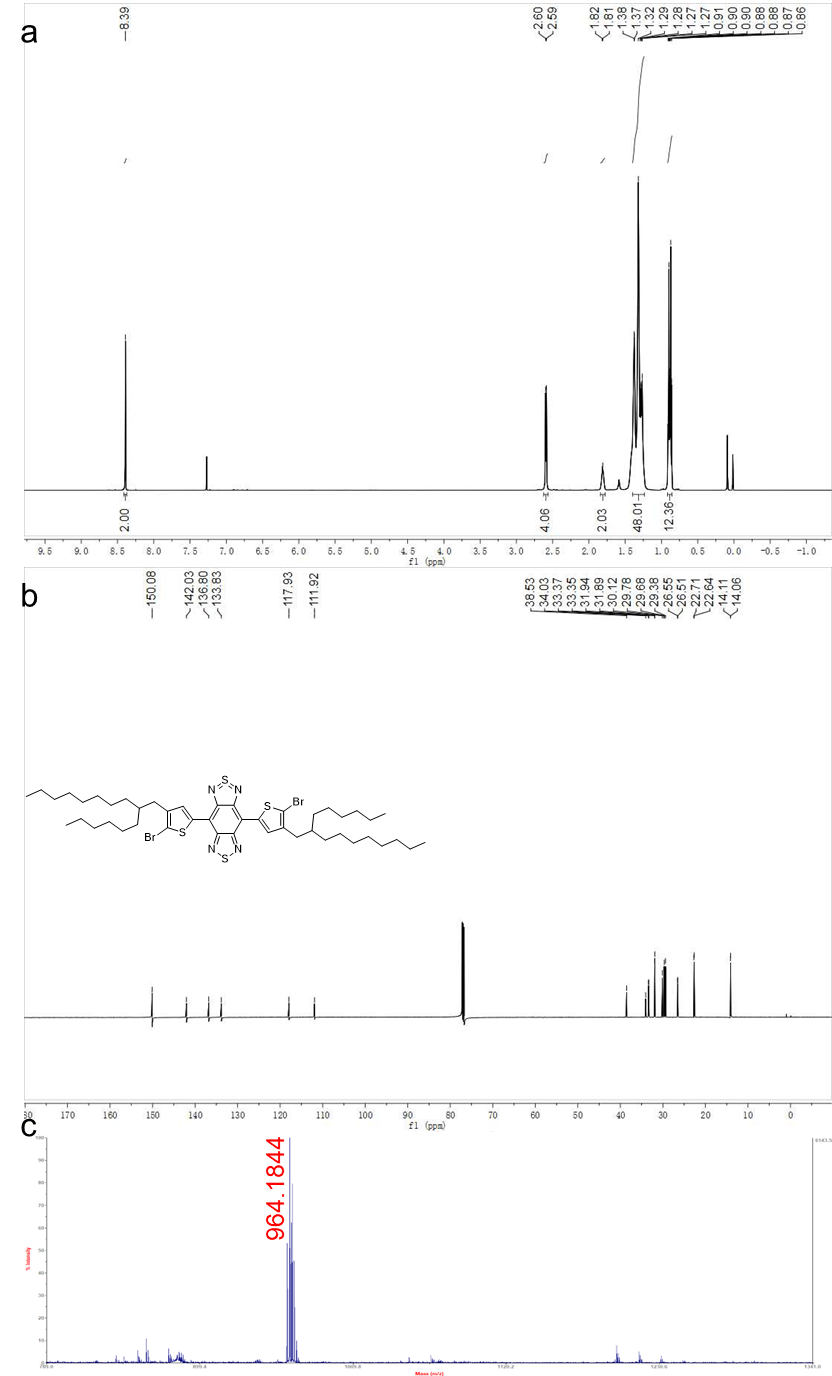


**Figure S6.** (a) ^1^H NMR spectra of Br-BBTPRO in CDCl_3_. (b) ^13^C NMR spectra of Br-BBTPRO in CDCl_3_. (c) MALDI-TOF (MS) spectrum of Br-BBTPRO.


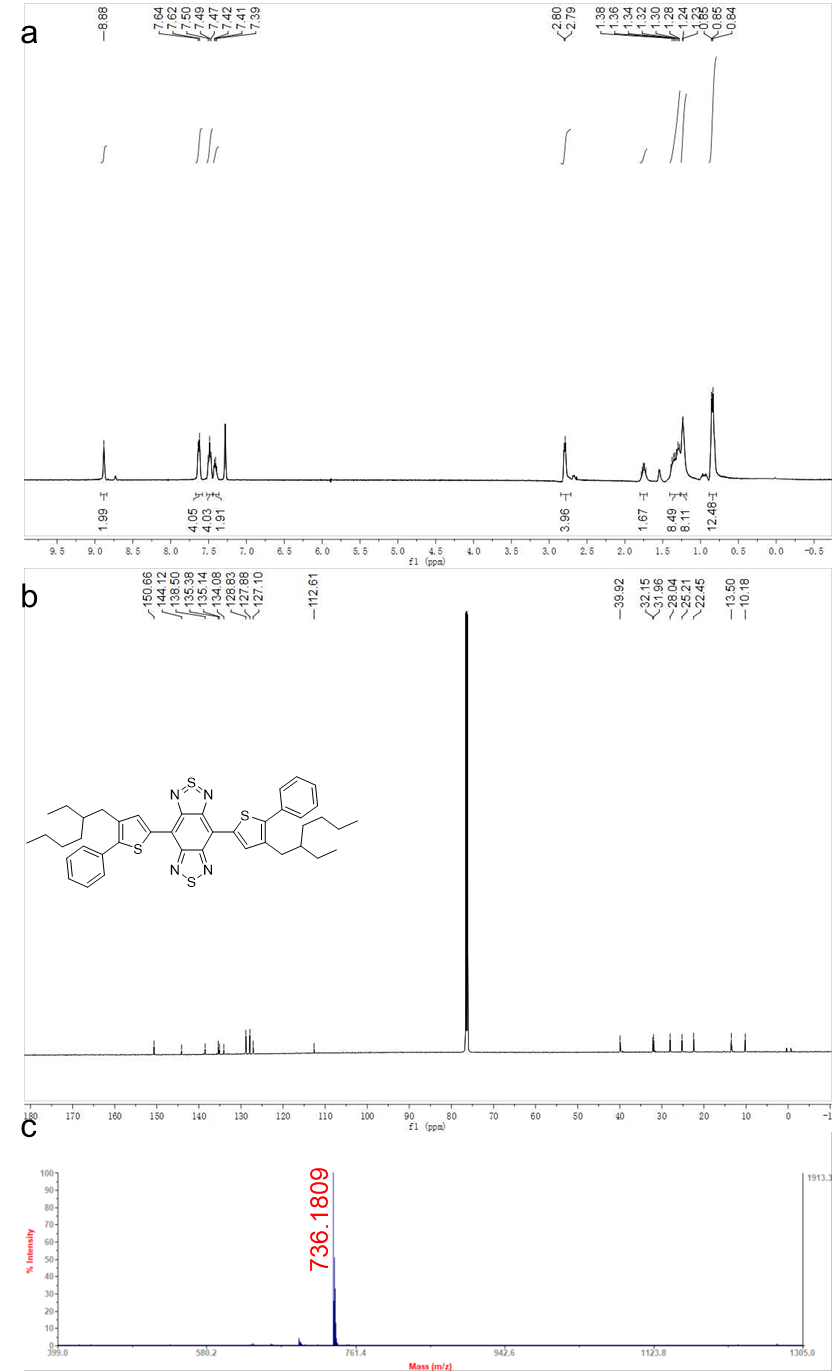


**Figure S7.** (a) ^1^H NMR spectra of BBTP in CDCl_3_. (b) ^13^C NMR spectra of BBTP in CDCl_3_. (c) MALDI-TOF (MS) spectrum of BBTP.


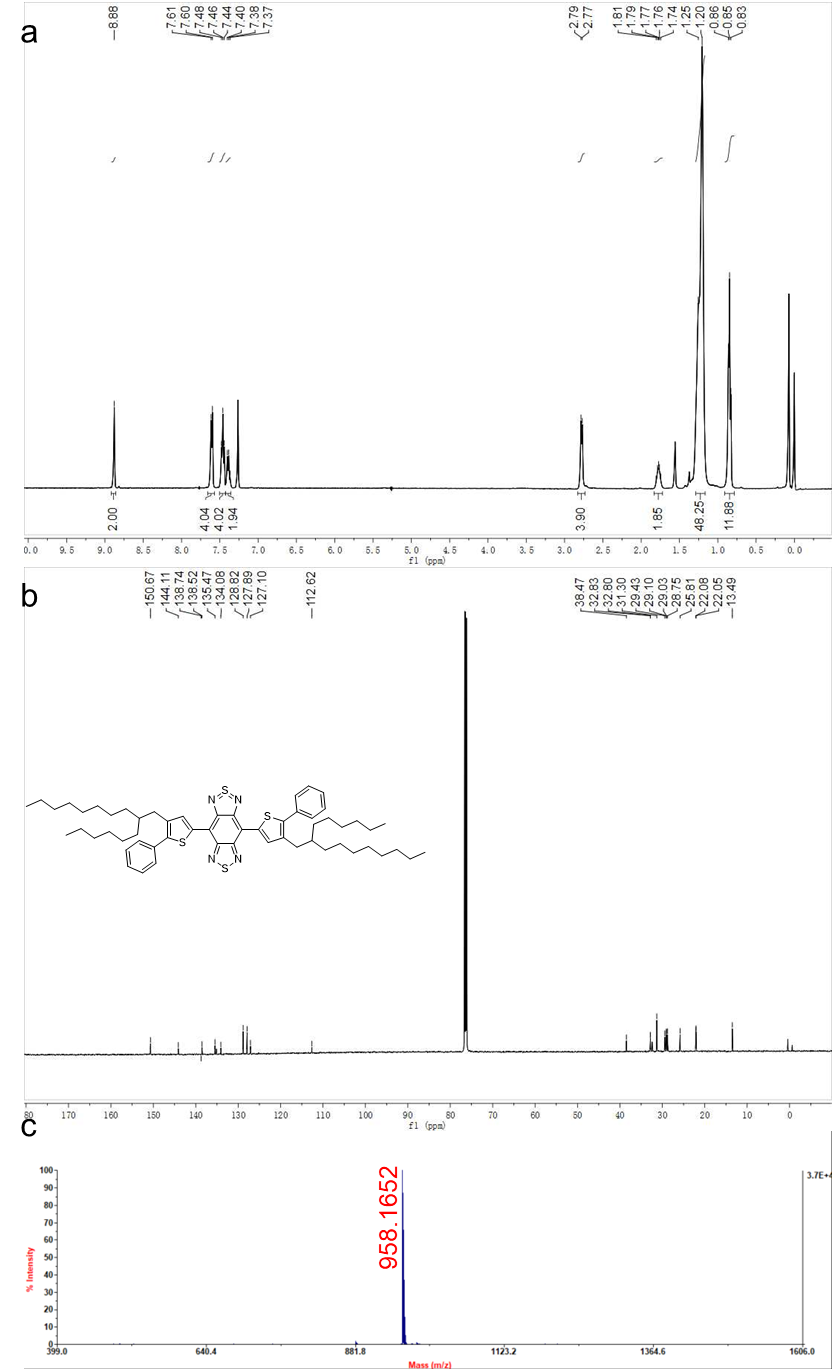


**Figure S8.** (a) ^1^H NMR spectra of BBTPPRO in CDCl_3_. (b) ^13^C NMR spectra of BBTPPRO in CDCl_3_. (c) MALDI-TOF (MS) spectrum of BBTPPRO.

**Figure S9.** Grazing-incidence wide-angle X-ray scattering (GIWAXS) spectra of substrate-free Raman molecules in solid state.

**Figure S10.** Plots of relative fluorescence Intensity (*I*/*I*_0_) in the H_2_O/THF mixtures with different water fractions. *I*_0_ and *I* represent the area under the curve of the fluorescence emission intensity of fluorophores in pure THF and different THF/water mixtures, respectively, λ_Ex_ at 808 nm, λ_Em_ ranged from 900 nm to 1300 nm.


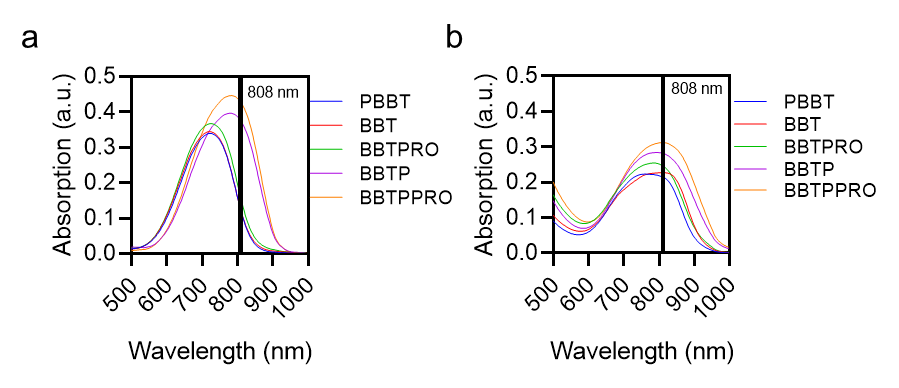


**Figure S11.** UV-Visible absorption spectra of substrate-free Raman molecules. (a) UV-Visible absorption spectra of molecules (50 μM) in THF. (b) UV-Visible absorption spectra of molecules (50 μM) in H_2_O/THF (95:5, v/v).


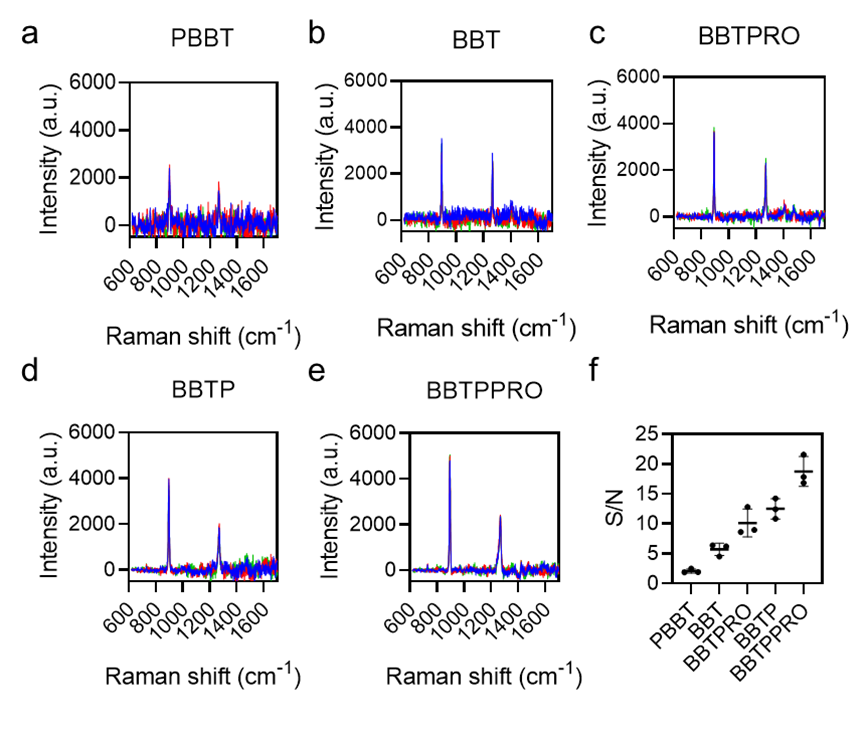


**Figure S12.** (a-e) Raman spectra of PBBT, BBT, BBTPRO, BBTP or BBTPPRO (10 μM) in H_2_O/THF (95:5, v/v) excited by 785 nm. Data are mean ± SD (*n* = 3). (f) The S/N of PBBT, BBT, BBTPRO, BBTP or BBTPPRO at 894 cm⁻^1^. Data are mean ± SD (*n* = 3).


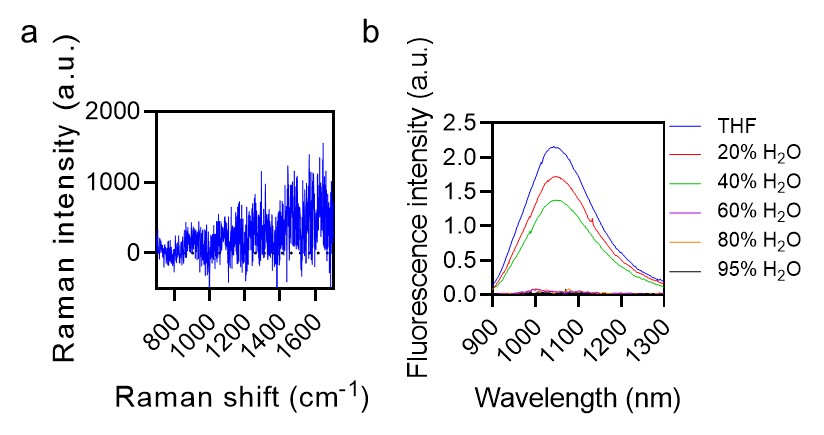


**Figure S13.** (a) Raman spectrum of BBTPPRO (20 μM) in THF after baseline correction. No peak at 894 cm^−1^. Raman measurement was carried out with a 785-nm laser excitation. (b) Fluorescence spectra of BBTPPRO (10 μM) in THF or the THF/water mixtures with THF fraction from 5% to 100%. λ_Ex_ at 808 nm, λ_Em_ ranged from 900 nm to 1300 nm.

.


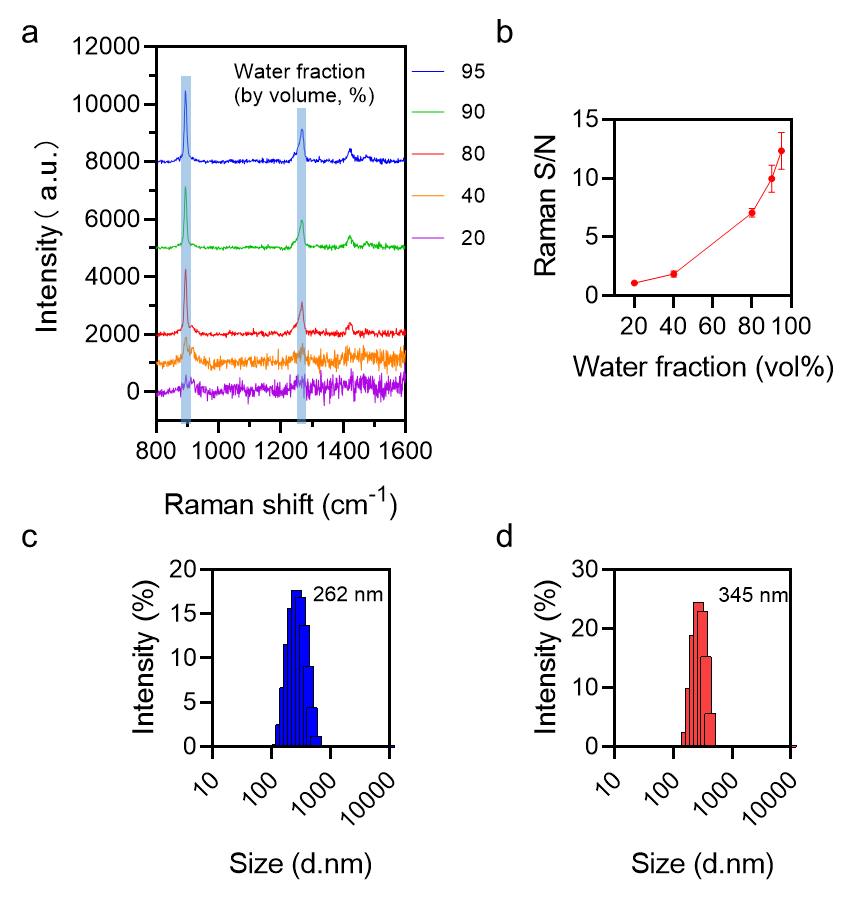


**Figure S14.** (a) Raman spectra of BBTPPRO (20 μM) in H_2_O/THF mixture. (b) Plot of S/N of Raman intensity at 894 cm^−1^ versus the water fraction of the H_2_O/THF mixtures in (a). When the water fraction was 20% and 40%, BBTPPRO did not show signal peak at 894 cm^−1^. Raman measurement was carried out with a 785-nm laser excitation. Blue columns in panel (a) represent the featured Raman peaks at 894 and 1,264 cm^−1^. (c, d) Size distributions of BBTPPRO in the mixture with a water fraction of 80% (c) and 95% (d) analyzed by dynamic light scattering, respectively.


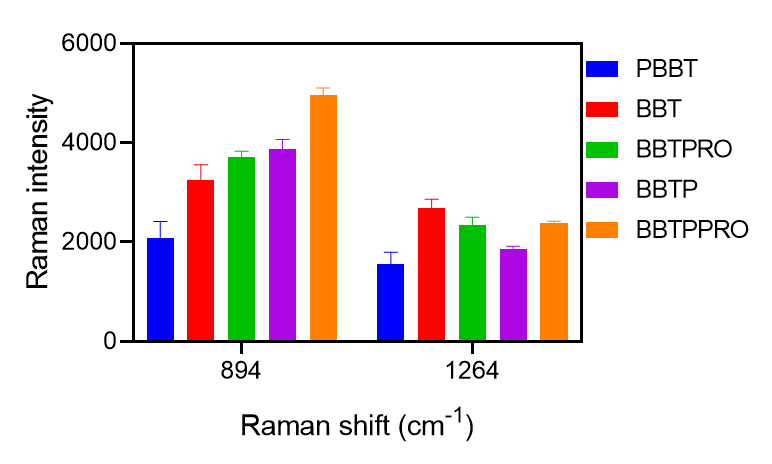


**Figure S15.** Raman intensity at 894 cm^−1^ and 1264 cm^−1^ of different molecules (10 μM) measured in the mixture of H_2_O/THF (95:5, v/v) with 785-nm excitation, 5 × objective, 1 s of acquisition time, and 5 times accumulation.


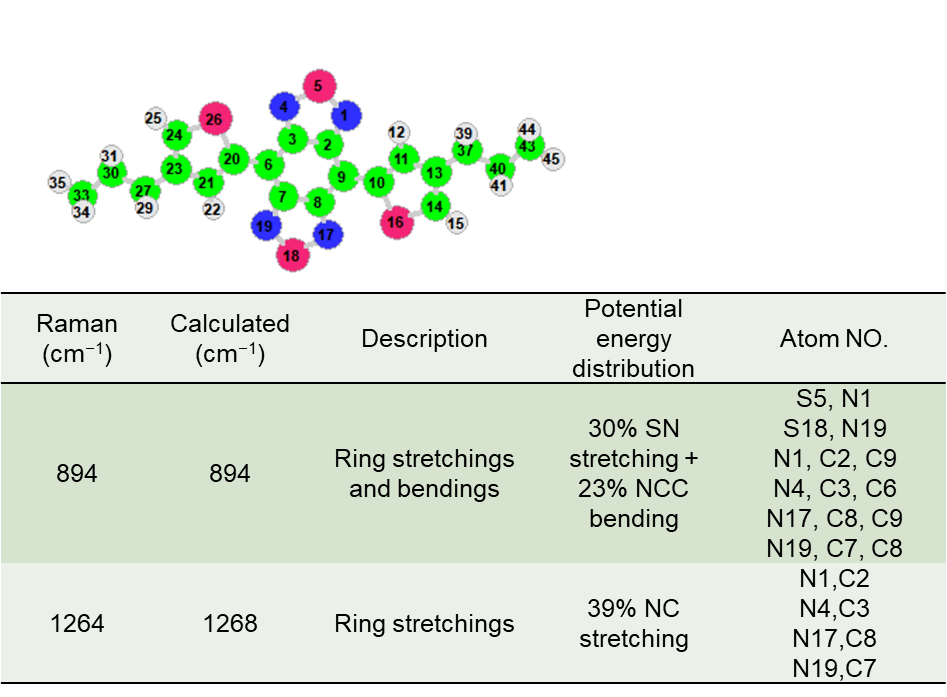


**Figure S16.** Experimental and calculated vibrational frequencies (cm^−1^) of PBBT and the corresponding assignments by DFT (Gaussian 09/B3LYP/6-31G (d)).


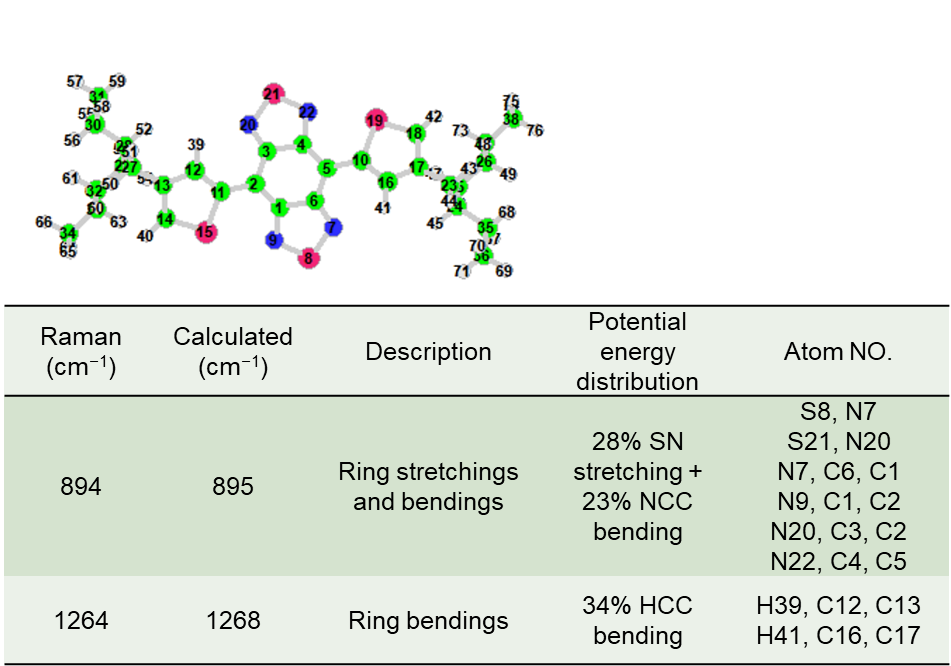


**Figure S17.** Experimental and calculated vibrational frequencies (cm^−1^) of BBT and the corresponding assignments by DFT (Gaussian 09/B3LYP/6-31G (d)).


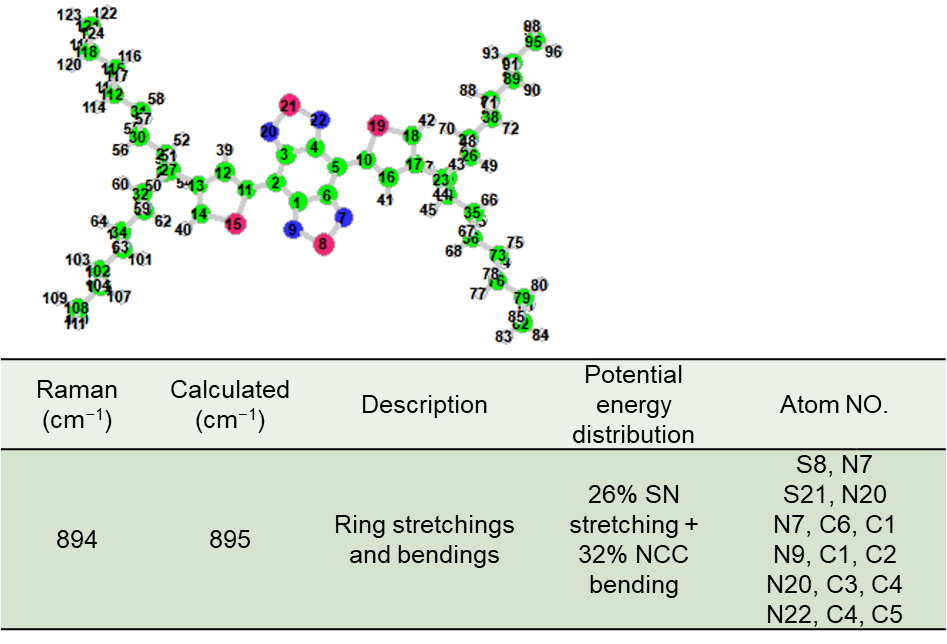


**Figure S18.** Experimental and calculated vibrational frequencies (cm^−1^) of BBTPRO and the corresponding assignments by DFT (Gaussian 09/B3LYP/6-31G (d)).


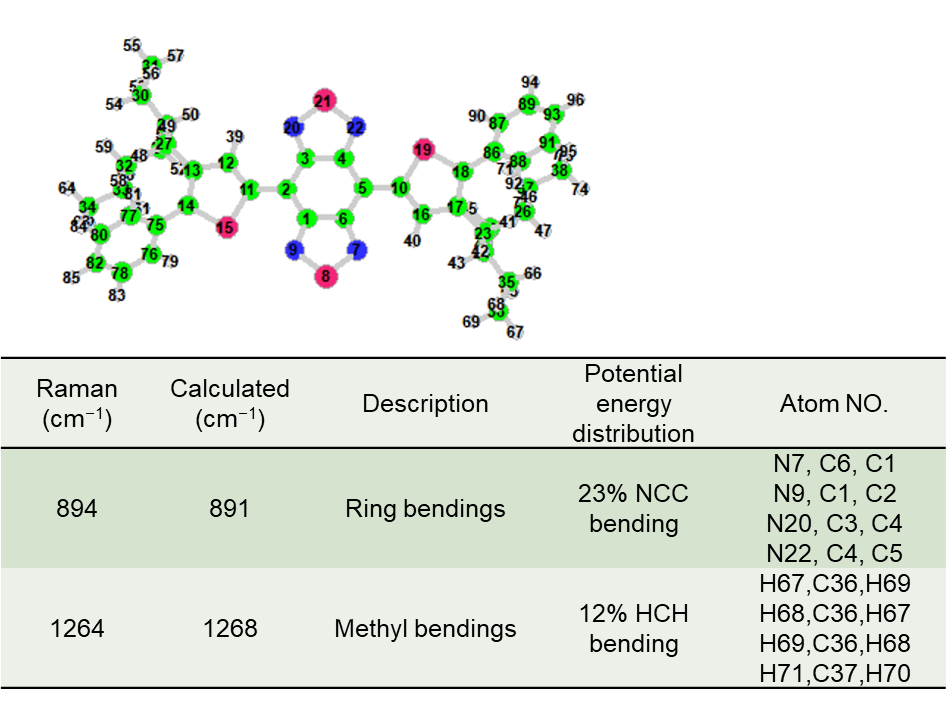


**Figure S19.** Experimental and calculated vibrational frequencies (cm^−1^) of BBTP and the corresponding assignments by DFT (Gaussian 09/B3LYP/6-31G (d)).


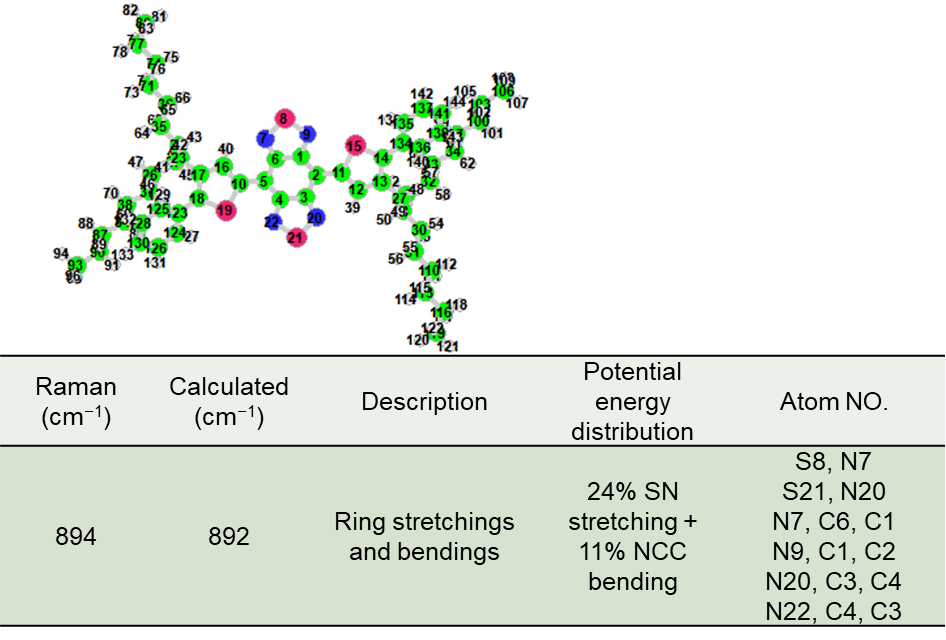


**Figure S20.** Experimental and calculated vibrational frequencies (cm^−1^) of BBTPPRO and the corresponding assignments by DFT (Gaussian 09/B3LYP/6-31G (d)).


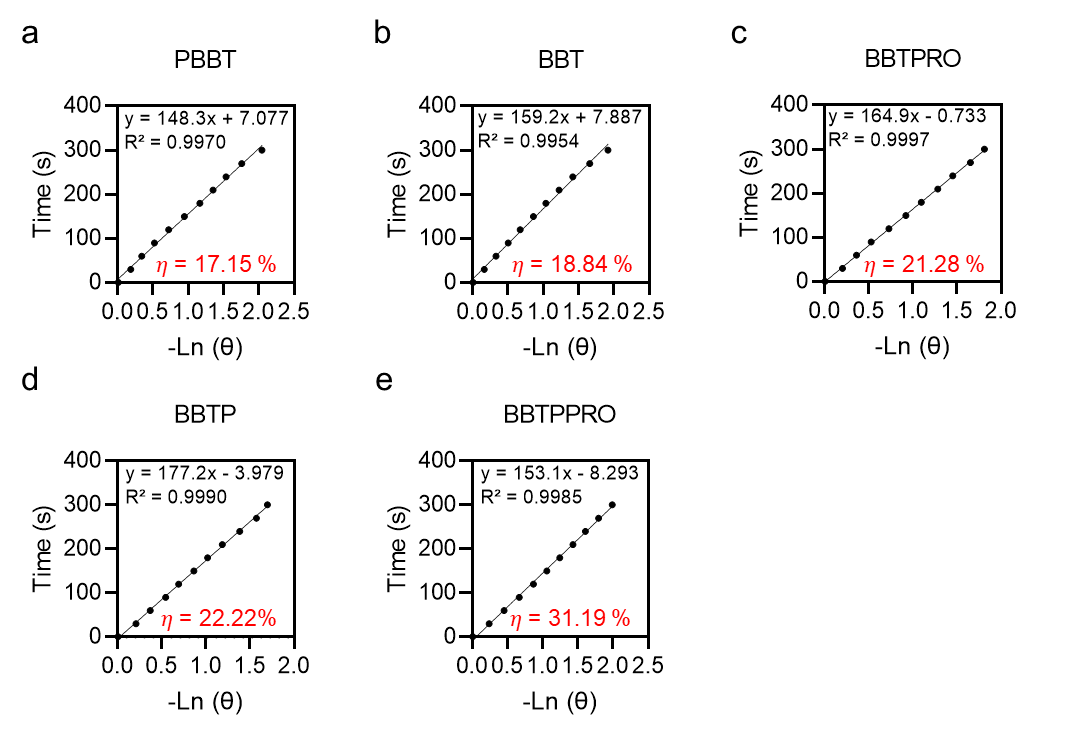


**Figure S21.** (a-e) Linear fitting of time from the cooling period versus the negative natural logarithm of driving force temperature for different substrate-free Raman molecules in H_2_O/THF (95:5, v/v).

**Figure S22.** Raman spectra of BBTPPRO NPs with BBTPPRO/DSPE-PEG ratios (mol/mol = 1:0.67) or BBTPPRO in the aggregates in the H_2_O/THF (95: 5, v/v) mixture. Raman measurement was carried out with a 785-nm laser excitation.


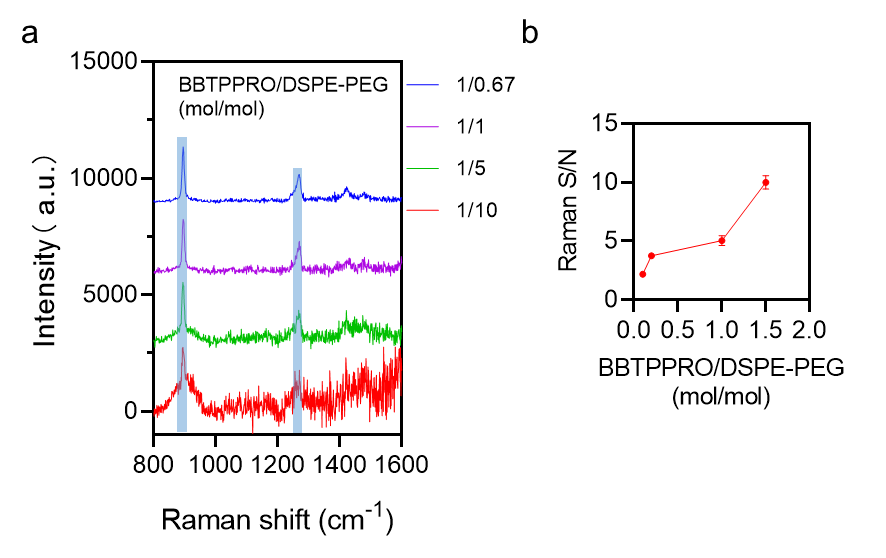


**Figure S23.** (a) Raman spectra of BBTPPRO NPs (20 μM) with different BBTPPRO/DSPE-PEG ratios (mol/mol). (b) Plot of S/N of Raman intensity at 894 cm^−1^ in (a). Blue columns in panel (a) represent the featured Raman peaks at 894 and 1264 cm^−1^, respectively.

**Figure S24.** UV-Visible absorption spectra of BBTPPRO (50 μM) in THF or in H_2_O/THF (95:5, v/v). and BBTPPRO NPs (50 μM) in H_2_O.

**Figure S25.** Raman spectra of BBTPPRO NPs at different time under 785 nm laser irradiation.

**Figure S26.** Temperature elevations (ΔT) of BBTPPRO NPs at different concentration under 808 nm laser irradiation (1 W/cm^2^).


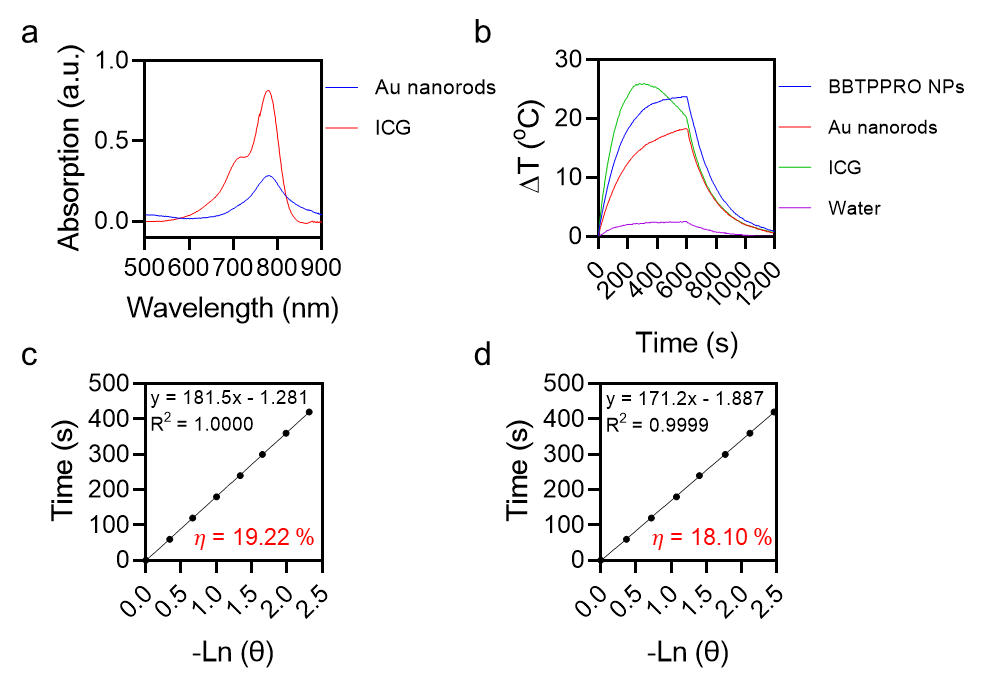


**Figure S27.** (a) UV-Visible absorption spectra of Au nanorods (8 μM of Au) and ICG (8 μM), respectively. (b) Temperature elevations (ΔT) of PTT agent solution (50 μM) under the laser irradiation (808 nm, 1 W/cm^2^) for 10 min, followed by another 10 min of cooling. (c) Linear fitting of time from the cooling period versus the negative natural logarithm of driving force temperature for Au nanorods. (d) Linear fitting of time from the cooling period versus the negative natural logarithm of driving force temperature for ICG.


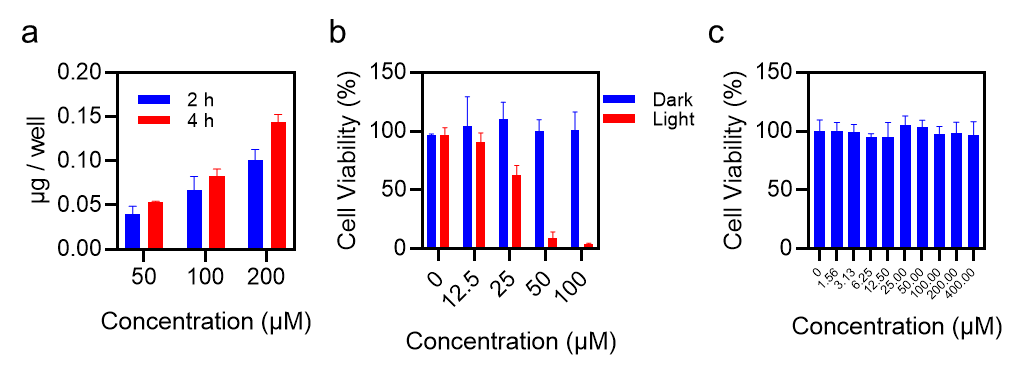


**Figure S28.** Cellular uptake, phototoxicity and toxicity of BBTPPRO NPs *in vitro*. (a) Uptake of BBTPPRO NPs by CT26-Luc mouse colon cancer cells after incubation at different concentrations for 2 h or 4 h. Data are presented as mean ± SD (*n* = 3). (b) Cell viability of CT26-luc tumor cells with different concentrations of BBTPPRO NPs in the absence or presence of 808 nm laser irradiation (1 W/cm^2^) for 5 min. Data are presented as mean ± SD (*n* = 3). (c) Cell viability of NIH 3T3 cells after incubation with BBTPPRO NPs at various concentrations for 24 h measured by MTT assay. Data are presented as mean ± SD (*n* = 4).


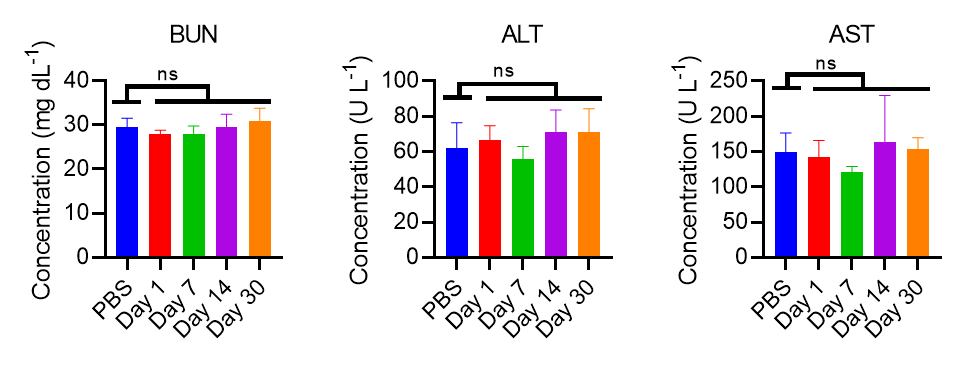


**Figure S29.** Serum biochemistry including blood urea nitrogen (BUN) levels and liver function markers such as alanine aminotransferase (ALT) and aspartate aminotransferase (AST) were measured. Serum samples were collected from ICR mice (male, 6−8 weeks, 20−22 g) at 1, 7, 14 or 30 d after i.v. injection of BBTPPRO NPs (20 mg/kg of BBTPPRO). Data are presented as mean ± SD (*n* = 3). Statistical significance was determined by one-way ANOVA with Tukey's multiple comparisons test. ns, no significant difference compared with PBS group.


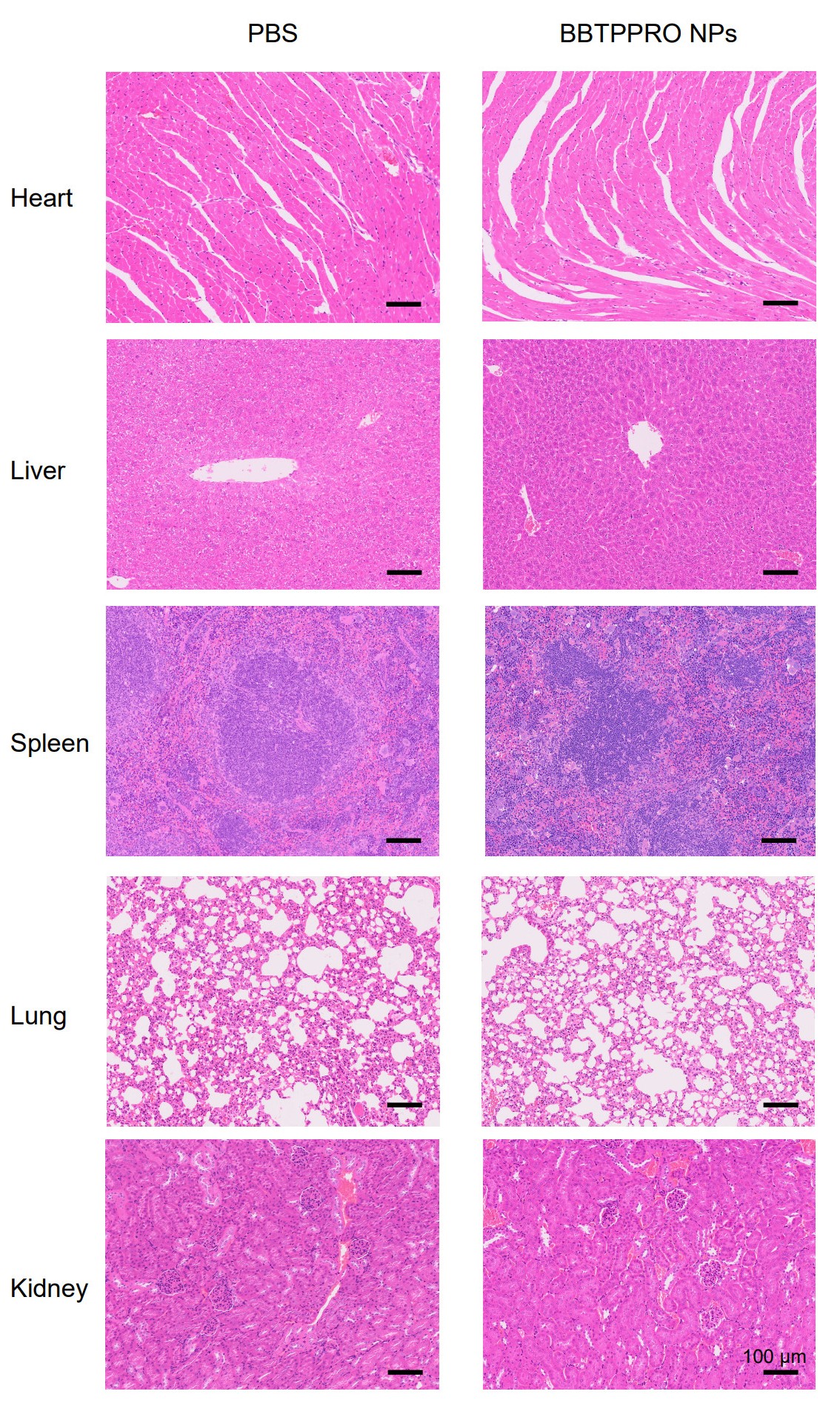


**Figure S30.** Histologic analysis with H&E staining of major organs of ICR mice at day 30 post-injection of BBTPPRO NPs (20 mg/kg) or PBS. Bar, 100 µm.


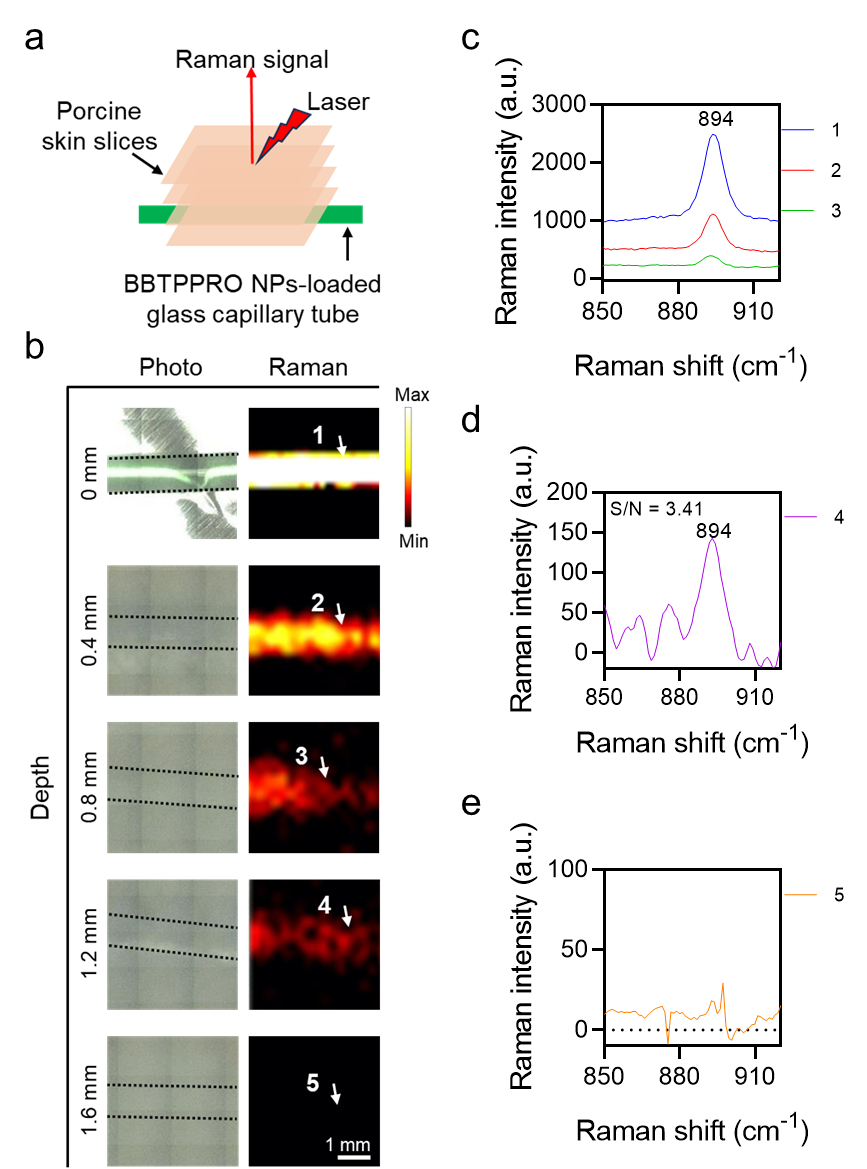


**Figure S31.** The SICTERS imaging of BBTPPRO NPs-loaded glass capillary tube covered with porcine skin slices. (a) Schematic diagram of the experimental setup. The thickness of each piece of porcine skin slices was ~0.4 mm. (b) The Raman imaging of the BBTPPRO NPs (1 mg/mL)-loaded glass capillary tube covered with or without porcine skin slices. (c) Raman spectra of Regions 1, 2, 3 in (b) of 0.0 mm, 0.4 mm, 0.8 mm in depth, respectively. (d) Raman spectrum of Region 4 in (b) of 1.2 mm in depth. (e) Raman spectrum of Region 5 in (b) of 1.6 mm in depth. The spectrum did not show peak at 894 cm^−1^. Raman measurement was carried out with 785-nm laser excitation.

**Figure S32.** Biodistribution profile of BBTPPRO in orthotopic CT26-Luc tumor-bearing mice at 1 h or 24 h after i.v. injection of BBTPPRO NPs (20 mg/kg). ID%/g tissue, percentage of the injected dose per gram tissue. Data are presented as mean ± SD (*n* = 5).


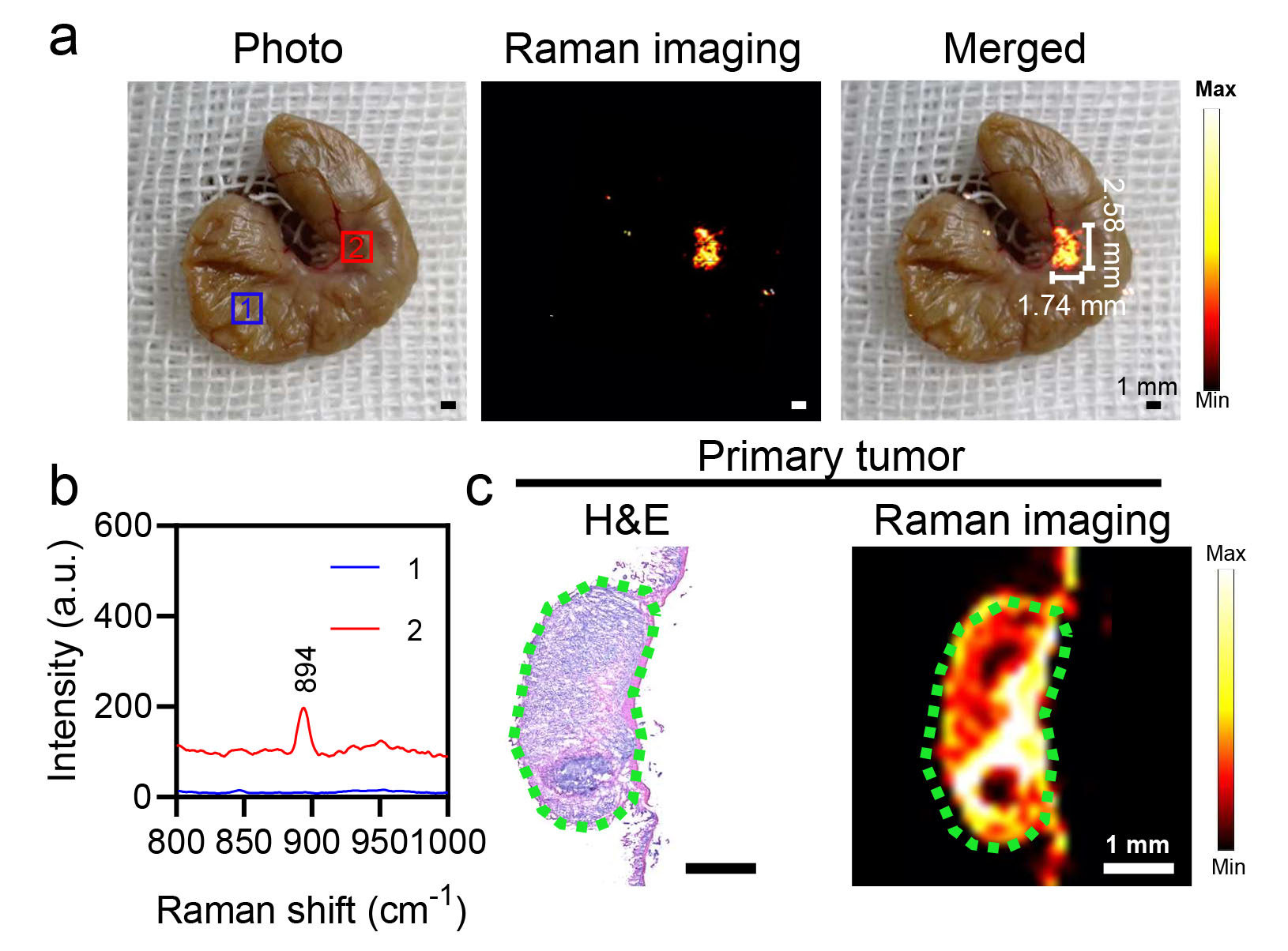


**Figure S33.** Intraoperative Raman imaging of orthotopic CT26-Luc mouse colon tumor by BBTPPRO NPs. (a) Intraoperative Raman imaging (894 cm^−1^) of primary lesion of mice following the i.v. injection of BBTPPRO NPs. Region 1, cecum tissue. Region 2, primary tumor. (b) Raman spectra of regions in (a), respectively. (c) Histological analysis and Raman imaging (894 cm^−1^) of primary tumor sections. Green circles, tumor.


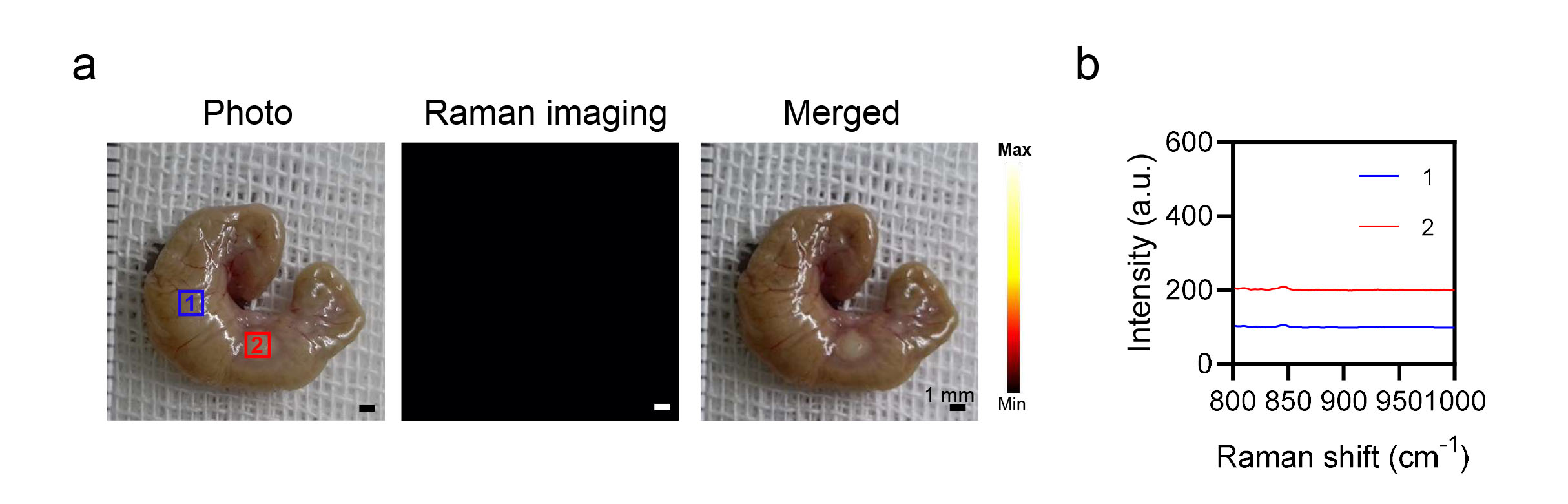


**Figure S34.** Intraoperative Raman imaging of orthotopic CT26-Luc mouse colon tumor with PBS. (a) Intraoperative Raman imaging (894 cm^−1^) of primary lesion of mice following the i.v. injection of PBS. Region 1, cecum tissue. Region 2, primary tumor. (b) Raman spectra of regions in (a), respectively, without showing peak at 894 cm^−1^.


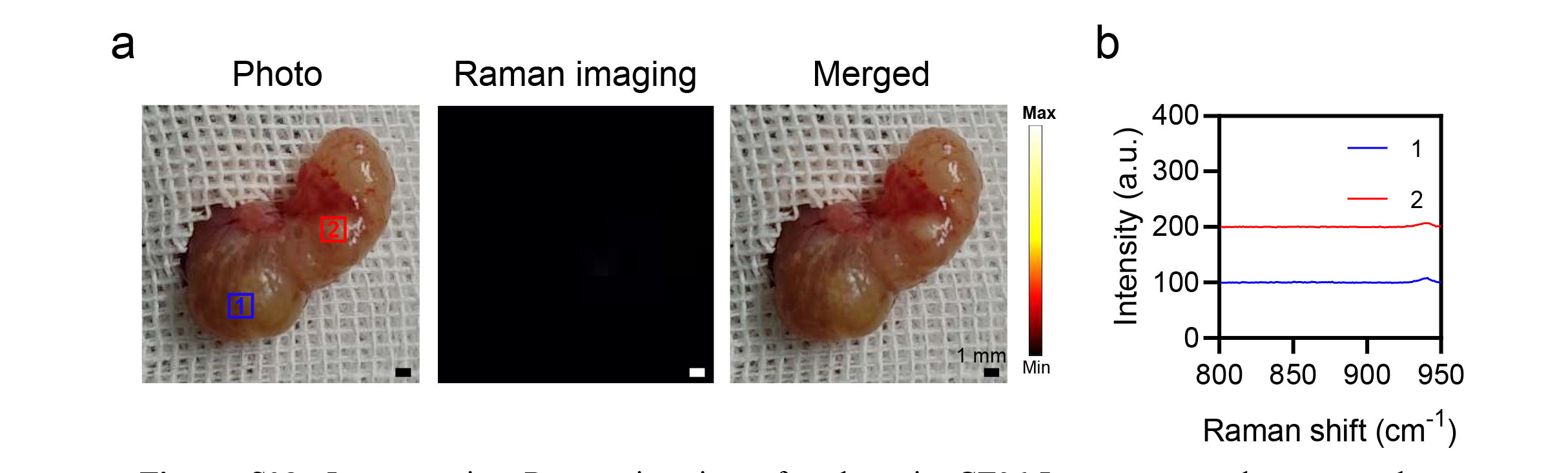


**Figure S35.** Intraoperative Raman imaging of orthotopic CT26-Luc mouse colon tumor by BBTPPRO@Au NPs. (a) Intraoperative Raman imaging (894 cm^−1^) of primary lesion of mice following the i.v. injection of BBTPPRO@Au NPs (7.5 mg/kg of Au). Region 1, cecum tissue. Region 2, primary tumor. (b) Raman spectra of regions in (a), respectively, without showing peak at 894 cm^−1^.


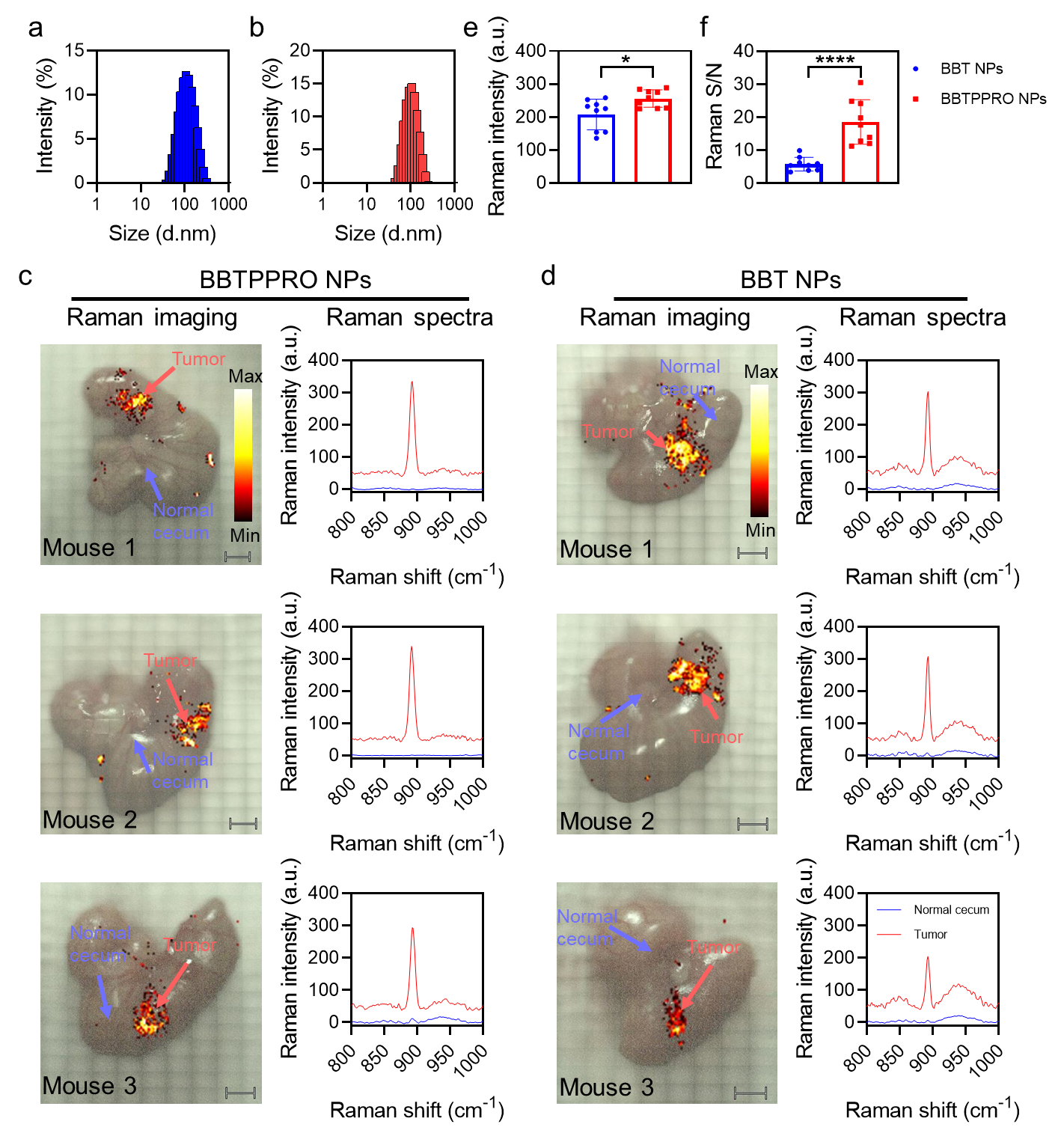


**Figure S36.** (a) Size distribution of BBT NPs. (b) Size distribution of BBTPPRO NPs. (c) Intraoperative Raman imaging of orthotopic CT26-Luc mouse colon tumor by BBTPPRO NPs (10 mg/kg). Raman imaging was performed on three mice separately. Left, intraoperative Raman imaging (894 cm^−1^) of tumor lesions. Right, Raman spectra of colon tumor (red curve), and normal cecum tissue (blue curve), respectively. Normal cecum showing no peak at 894 cm^−1^. Bar, 2 mm. (d) Intraoperative Raman imaging of orthotopic CT26-Luc mouse colon tumor by BBT NPs (10 mg/kg). Raman imaging was performed on three mice separately. Left, intraoperative Raman imaging (894 cm^−1^) of tumor lesions. Right, Raman spectra of colon tumor (red curve), and normal cecum tissue (blue curve), respectively. Normal cecum showing no peak at 894 cm^−1^. Bar, 2 mm. (e) Raman intensity at 894 cm^−1^ of tumor lesions in BBT NPs group, or BBTPPRO NPs group. Three tumor of mice each group with three sites per tumor were analyzed. Data are mean ± SD (*n* = 9). Statistical significance was calculated via unpaired t test. **p* < 0.05, compared BBT NPs group with BBTPPRO NPs group. (f) Raman signal to noise ratio (S/N) at 894 cm^−1^ of tumor lesions in BBT NPs group, or BBTPPRO NPs group. Three tumor of mice each group with three sites per tumor were analyzed. Data are mean ± SD (*n* = 9). Statistical significance was analyzed via unpaired *t* test. *****p* < 0.0001, compared BBT NPs group with BBTPPRO NPs group.


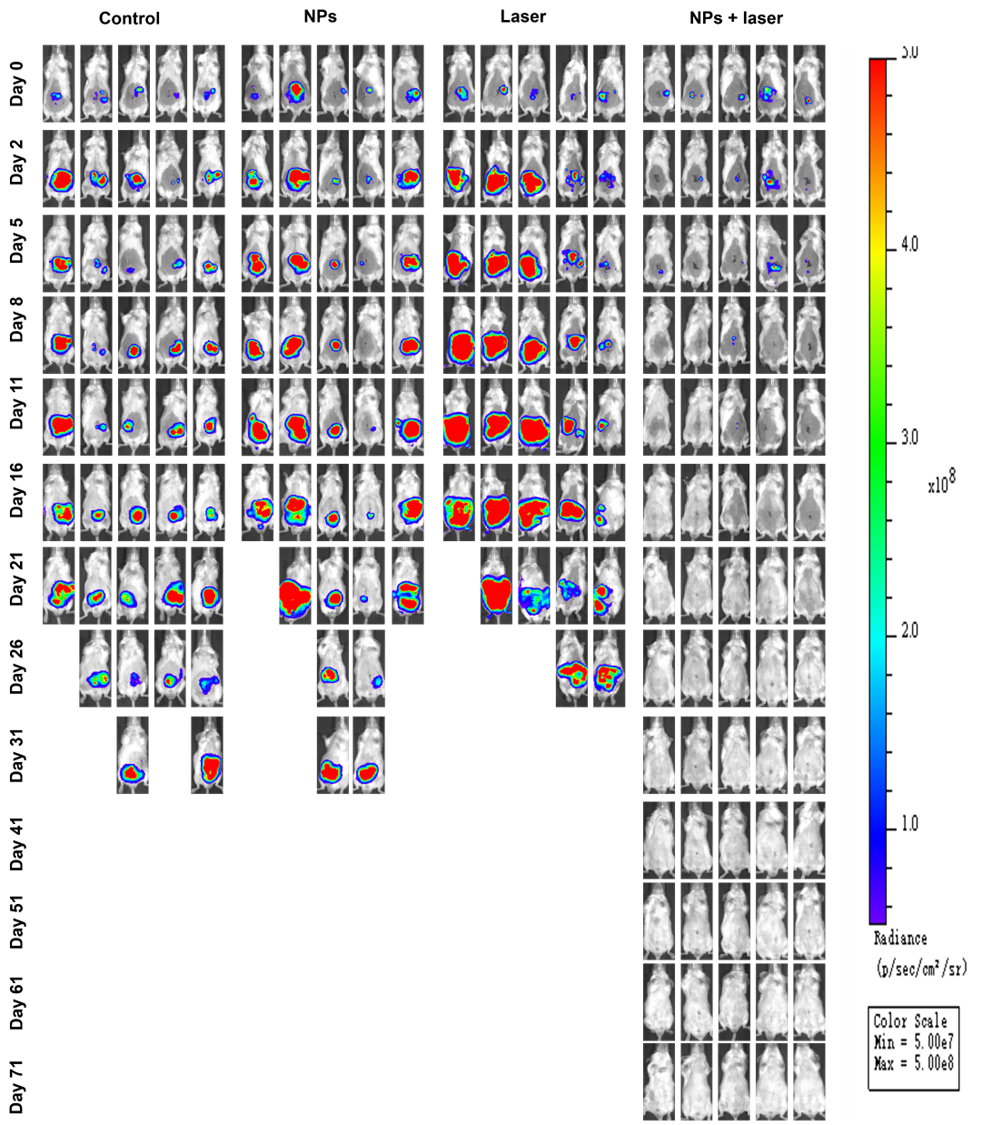


**Figure S37.** *In vivo* bioluminescence imaging of CT26-Luc tumors before (Day 0) or after different treatment (*n =* 5). In Control group, mice received i.v. injection of PBS without other treatment. In NPs group, mice received i.v. injection of BBTPPRO NPs (7.5 mg/kg) without other treatment. In Laser group, mice received i.v. injection of PBS plus laser irradiation (808 nm, 1 W/cm^2^, 8 min). The primary lesion was visualized by eye intraoperatively. In NPs + Laser group, mice received i.v. injection of BBTPPRO NPs (7.5 mg/kg). After 24 h, primary and metastatic tumor was visualized under intraoperative Raman imaging followed by PTT (808 nm, 1 W/cm^2^, 5 min for primary tumor; 1 W/cm^2^, 8 min for metastatic tumor). In both laser-treated groups, the cecum was repositioned in the abdominal cavity following PTT. The skin was closed with suture.


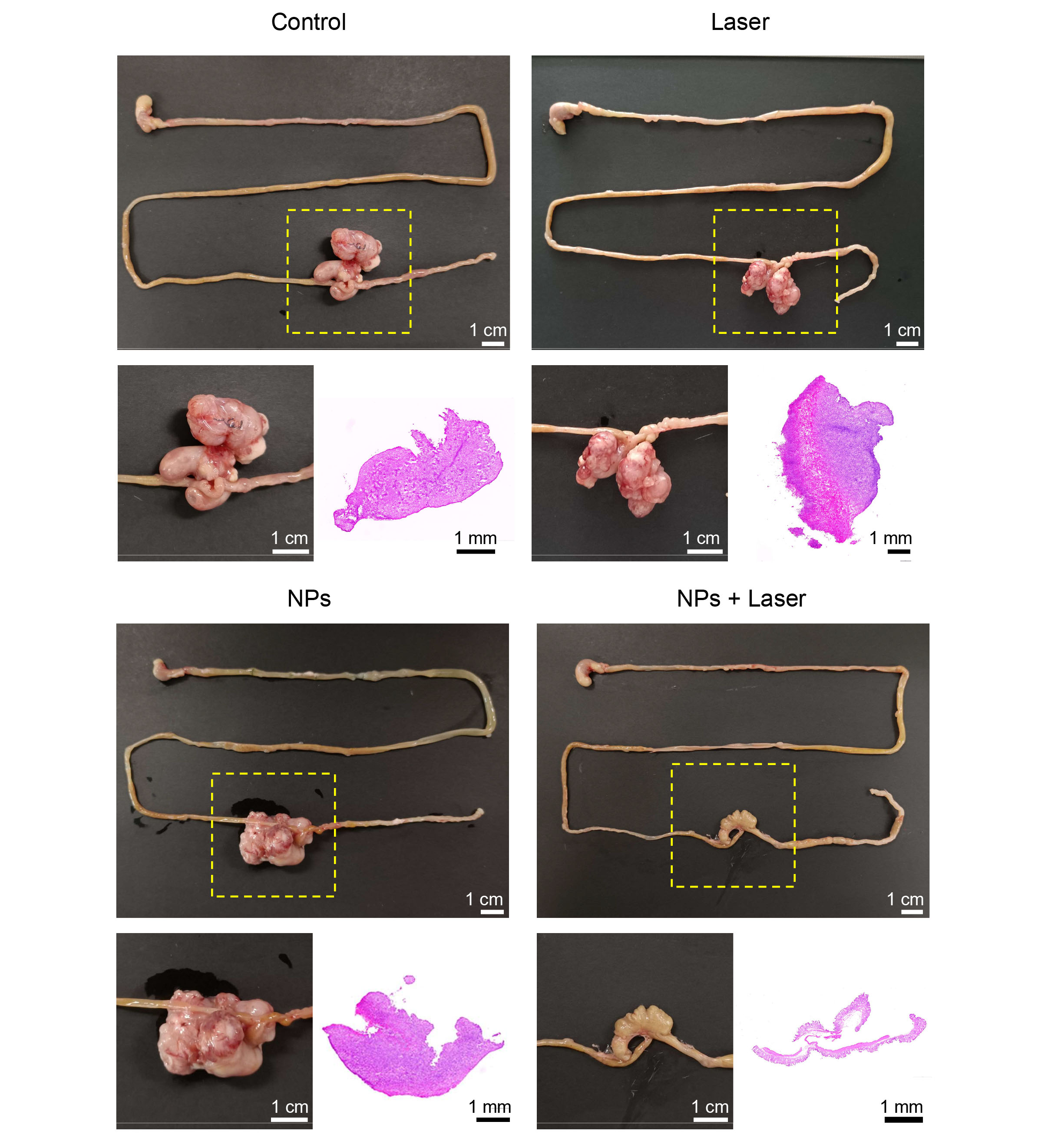


**Figure S38.** Representative photographs of the resected gastrointestinal tract and histological examination of the tumor or cecum of the mouse with H&E staining at the end of each experiment following the treatment in Figure S37.


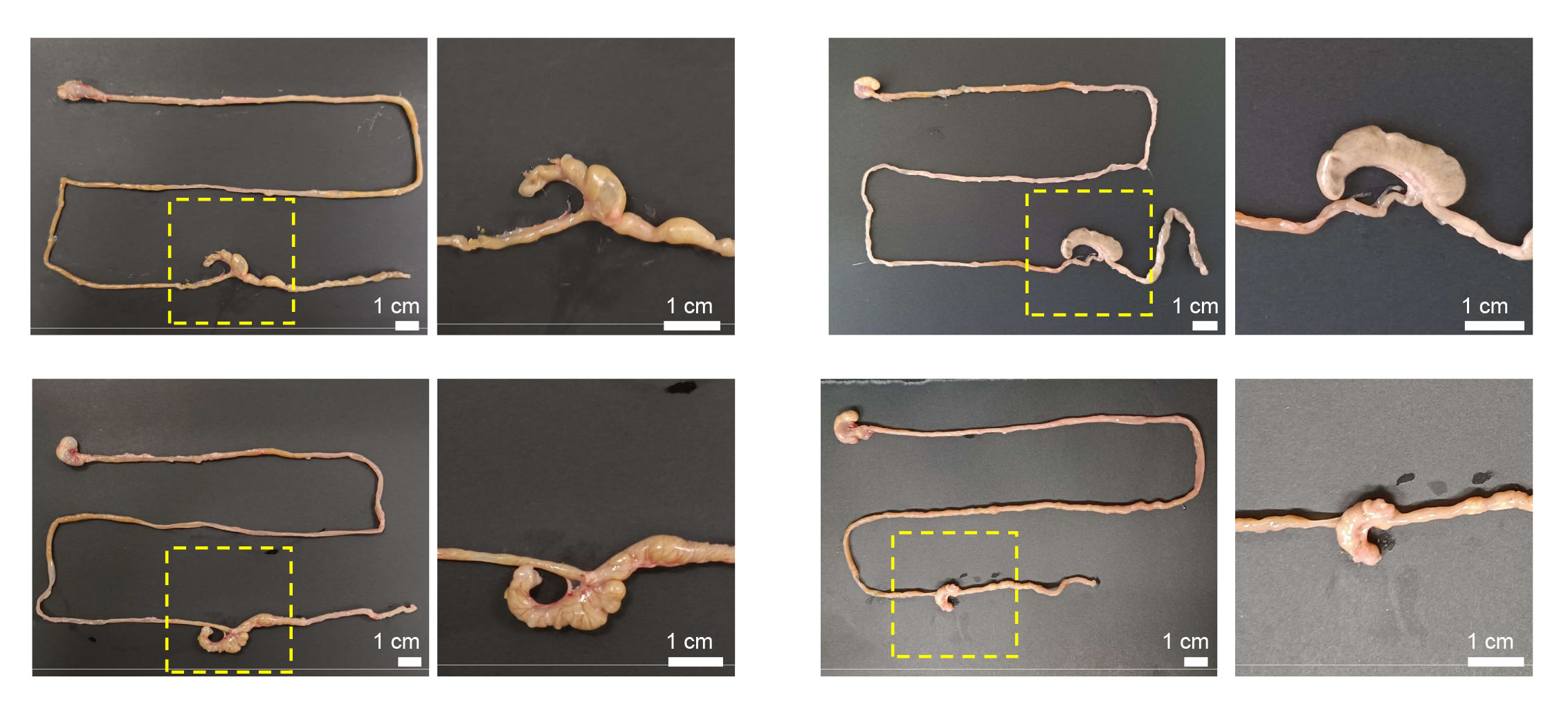


**Figure S39.** Photographs of the resected gastrointestinal tract including cecum of the other four mice in NPs + Laser group at Day 71 following the treatment in Figure S37.

**Table S1. Blood chemistry and hematologic analysis of mice.*^a^***

| Item | Reference range | Control | Day 1 | Day 7 | Day 14 | Day 30 |
| --- | --- | --- | --- | --- | --- | --- |
|  |  |  |  |  |  |  |
| WBC (10^9^ L^−1^) | 4.44-14.01 | 7.1 ± 1.1 | 7.5 ± 3.2 | 10.7 ± 3.0 | 12.4 ± 4.7 | 13.5 ± 2.4 |
| LYMPH (10^9^ L^−1^) | 2.06-10.01 | 4.9 ± 1.0 | 4.1 ± 1.4 | 6.8 ± 1.3 | 8.4 ± 4.6 | 8.2 ± 2.3 |
| MONO (10^9^ L^−1^) | 0.18-1.32 | 0.4 ± 0.3 | 0.6 ± 0.4 | 0.6 ± 0.5 | 0.7 ± 0.3 | 0.9 ± 0.6 |
| NEUT (10^9^ L^−1^) | 0.53-5.17 | 1.8 ± 0.9 | 2.8 ± 1.6 | 3.3 ± 2.1 | 3.3 ± 1.1 | 4.4 ± 0.5 |
| LYMPH (%) | 37.50-85.01 | 68.8 ± 13.9 | 57.2 ± 15.2* | 64.8 ± 13.7 | 65.2 ± 12.5 | 60.1 ± 8.2 |
| MONO (%) | 2.84-13.09 | 6.1 ± 4.1 | 7.9 ± 4.3 | 5.7 ± 3.2 | 6.8 ± 4.4 | 6.9 ± 4.7 |
| NEUT (%) | 8.74-55.68 | 25.1 ± 9.8 | 34.9 ± 11.1 | 29.6 ± 10.5 | 28.0 ± 9.0 | 33.0 ± 4.2 |
| RBC (10^12^ L^−1^) | 7.31-12.27 | 9.1 ± 0.2 | 10.0 ± 0.7 | 10.2 ± 1.5 | 8.8 ± 1.5 | 10.3 ± 0.7 |
| HGB (g dL^−1^) | 11.9-18.4 | 19.0 ± 1.5 | 21.6 ± 3.5 | 20.8 ± 2.8 | 25.2 ± 6.1 | 19.7 ± 1.9 |
| HCT (%) | 39.7-74.7 | 46.1 ± 1.5 | 51.2 ± 5.4 | 50.9 ± 6.7 | 46.2 ± 8.8 | 48.9 ± 3.1 |
| MCV (fL) | 46.5-69.0 | 50.9 ± 1.6 | 51.2 ± 1.9 | 50.1 ± 0.9 | 52.6 ± 1.1 | 47.7 ± 0.4 |
| MCH (pg) | 13.1-18.0 | 20.9 ± 2.0 | 21.7 ± 4.3 | 20.4 ± 0.4 | 29.0 ± 7.9 | 19.3 ± 3.2 |
| MCHC (g dL^−1^) | 21.3-33.9 | 41.2 ± 3.0 | 42.5 ± 8.3 | 40.7 ± 0.9 | 55.3 ± 15.3 | 40.6 ± 6.6 |
| RDW (%) | 15.1-18.9 | 15.7 ± 0.4 | 16.9 ± 0.5 | 16.4 ± 0.6 | 16.1 ± 0.8 | 15.0 ± 0.6 |
| PLT (10^9^ L^−1^) | 736-2374 | 1331.0 ± 308.4 | 607.0 ± 94.4 | 1091.3 ± 189.2 | 561.3 ± 206.2 | 702.0 ± 195.9 |
| MPV (fL) | 4.3-5.8 | 5.7 ± 0.5 | 6.1 ± 0.2 | 5.5 ± 0.3 | 6.6 ± 0.9 | 5.8 ± 0.3 |

***^a^*** Blood samples were collected from ICR mice (male, 6−8 weeks, 20−22 g) at 1, 7, 14 or 30 d after i.v. injection of BBTPPRO NPs (20 mg/kg of BBTPPRO). Mice without treatment were used as control. Complete blood counts: blood levels of white blood cells (WBC), lymphocytes (LYMPH), monocytes (MONO), neutrophils (NEUT), red blood cells (RBC), hemoglobin (HGB), hematocrit (HCT), mean corpuscular volume (MCV), mean corpuscular hemoglobin (MCH), mean corpuscular hemoglobin concentration (MCHC), red cell volume distribution width (RDW), platelets (PLT), mean platelet volume (MPV). Reference ranges of hematology data of healthy male ICR mice were obtained from Charles River Laboratories: (http://www.criver.com/). Data are presented as mean ± SD (*n* = 3). Statistical significance was determined by two-way ANOVA with Tukey's multiple comparisons test. **p* < 0.05, compared with Control group.

**References**

(1) S. Gao, Y. Zhang, K. Cui, S. Zhang, Y. Qiu, Y. Liao, H. Wang, S. Yu, L. Ma, H. Chen, M. Ji, X. Fang, W. Lu, Z. Xiao, Self-stacked Small Molecules for Ultrasensitive, Substrate-free Raman Imaging *In Vivo*. *Nat Biotechnol* **2024**, *43*, 936.

(2) S. Gao, G. Wei, S. Zhang, B. Zheng, J. Xu, G. Chen, M. Li, S. Song, W. Fu, Z. Xiao, W. Lu, Albumin Tailoring Fluorescence and Photothermal Conversion Effect of Near-Infrared-II Fluorophore with Aggregation-Induced Emission Characteristics. *Nat Commun* **2019**, *10*, 2206.

**Supplementary Movies**

**Movie S1**: The ring stretching and bending modes of the multi-ring skeleton of BBT at peak 894 cm^−1^.

**Movie S2**: The ring stretching and bending modes of the multi-ring skeleton of BBTP at peak 894 cm^−1^.

**Movie S3**: The ring stretching and bending modes of the multi-ring skeleton of BBTPPRO at peak 894 cm^−1^.

**Movie S4**: The ring stretching and bending modes of the multi-ring skeleton of BBTPRO at peak 894 cm^−1^.

**Movie S5**: The ring stretching and bending modes of the multi-ring skeleton of PBBT at peak 894 cm^−1^.
